# Short‐Chain Fatty Acids: Microbial Metabolites Driving Gut‐Eye Axis Signaling

**DOI:** 10.1002/cph4.70251

**Published:** 2026-09-09

**Authors:** Aleah J. Brokemond, Ronny Amamoo, Fapianey J. Alexandre, Sewedo B. Ajisegiri, Menaka C. Thounaojam, Pamela M. Martin, Ravirajsinh N. Jadeja

**Affiliations:** ^1^ Department of Biomedical Sciences, School of Graduate Studies Meharry Medical College Nashville Tennessee USA

**Keywords:** butyrate, gut‐eye axis, microbiome, ocular diseases, short‐chain fatty acids

## Abstract

Short‐chain fatty acids (SCFAs) have emerged as key molecular mediators of gut–eye communication, linking microbial metabolism to ocular physiology and disease. Produced through microbial fermentation of dietary fiber, SCFAs function as systemic signaling molecules that regulate immune homeostasis, metabolic function, vascular integrity, and neuroinflammatory pathways through activation of SCFA‐responsive receptors and epigenetic mechanisms. Growing evidence indicates that SCFAs influence biological processes central to ocular health, including inflammation, oxidative stress, neurodegeneration, and pathological angiogenesis. Experimental, metabolomic, and emerging clinical studies suggest that disruptions in SCFA signaling may contribute to the pathogenesis of retinal and ocular surface diseases; however, the mechanisms governing ocular exposure, target engagement, and therapeutic efficacy remain incompletely understood, highlighting a critical gap between mechanistic insight and clinical translation. In this review, we integrate current evidence into a mechanistic and translational framework that positions SCFAs as central effectors of the gut–eye axis. We further evaluate the therapeutic potential of SCFA modulation and identify key barriers to clinical implementation, including bioavailability, pharmacokinetics, dosing, and long‐term safety, thereby delineating a roadmap for the development of microbiome‐derived precision therapeutics in ophthalmology.

## Graphical abstract

Short‐chain fatty acids generated by gut microbial fermentation link diet to ocular physiology via the gut–eye axis. Through systemic distribution, these metabolites regulate GPCR signaling, epigenetic pathways, inflammation, and angiogenesis, shaping retinal homeostasis and highlighting therapeutic potential in ocular vascular and inflammatory diseases. The illustration was created in BioRender.

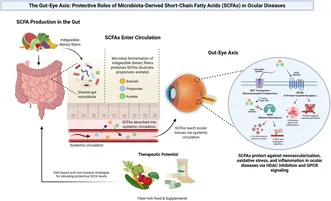

## 1 Introduction

The gut microbiome is increasingly recognized as a regulator of systemic physiology, largely through the production of bioactive metabolites that modulate immune, metabolic, vascular, and neuroinflammatory processes (Fusco et al. 2023; Mansuy‐Aubert and Ravussin 2023; Facchin et al. 2024). Beyond their local functions within the gastrointestinal tract, these metabolites mediate inter‐organ communication with distal tissues, forming the basis of axes such as the gut–lung and gut–brain systems (Carabotti et al. 2015; Liu et al. 2026; Loh et al. 2024). More recently, this paradigm has been extended to the eye, giving rise to the concept of the gut–eye axis (Zhou et al. 2025; Szymanska et al. 2026; Gao et al. 2026). Alterations in gut microbial composition have been associated with several ocular disorders, including diabetic retinopathy (DR), age‐related macular degeneration (AMD), glaucoma, and immune‐mediated ocular inflammation (Wang et al. 2026; Wang et al. 2025; Suresh et al. 2026; Sadeghi et al. 2026). However, the molecular signals linking gut microbial activity to ocular physiology remain incompletely defined.

Among microbiome‐derived metabolites, short‐chain fatty acids (SCFAs) are among the best characterized due to their established roles in systemic signaling and host physiology (Oxenrider et al. 2025; Parker et al. 2022). SCFAs are saturated fatty acids containing fewer than six carbon atoms, with acetate (C2), propionate (C3), and butyrate (C4) representing the predominant forms generated through microbial fermentation of dietary fibers such as resistant starches, pectin, inulin, arabinoxylans, and β‐glucans (Campagnoli et al. 2023; Kammoun et al. 2024). Following their production in the colon, SCFAs are absorbed into the circulation and distributed systemically, where they influence multiple physiological systems (Chen et al. 2022).

SCFAs exert their biological effects through both receptor‐dependent and receptor‐independent mechanisms. They activate G protein‐coupled receptors (GPCRs), primarily free fatty acid receptor 2 (FFAR2/GPR43), free fatty acid receptor 3 (FFAR3/GPR41), and hydroxycarboxylic acid receptor 2 (HCAR2/GPR109A), and regulate gene expression through inhibition of histone deacetylases (HDACs) (Facchin et al. 2024; Mann et al. 2024). Through these pathways, SCFAs influence immune cell differentiation, cytokine production, epithelial barrier integrity, cellular metabolism, oxidative stress responses, and vascular homeostasis (Mann et al. 2024; Feng and Xu 2023; He et al. 2020; Liu et al. 2018; Mukhopadhya and Louis 2025; Nastasi et al. 2017; Zhang et al. 2023), positioning them as key systemic signals that link gut microbial activity to distal tissue function.

The eye, despite its immune‐privileged status and specialized barriers, is responsive to systemic metabolic and immune cues (He et al. 2020; Song et al. 2025). Processes regulated by SCFAs, including inflammation, oxidative stress, neurodegeneration, and pathological angiogenesis, are central to the pathogenesis of ocular disease. Experimental models have demonstrated protective effects of SCFAs in retinal neovascularization and ocular inflammation, while microbiome and metabolomic studies have identified associations between altered SCFA‐related pathways and retinal disorders (Oxenrider et al. 2025; Parker et al. 2022; Campagnoli et al. 2023; Kammoun et al. 2024; Adhikary et al. 2026; Rowan et al. 2017). Collectively, these findings position SCFAs as leading candidate effectors of the gut–eye axis and suggest a mechanistic basis through which microbial metabolism influences ocular homeostasis and disease susceptibility.

Despite these advances, critical questions remain. The extent to which circulating SCFAs reach ocular tissues, the mechanisms governing tissue‐specific signaling within the eye, and the relevance of experimental findings to human disease remain incompletely understood. Moreover, efforts to translate microbiome‐targeted and SCFA‐based interventions into clinical applications are constrained by challenges related to bioavailability, pharmacokinetics, dosing, and long‐term safety.

This review consolidates current evidence to establish a mechanistic and translational framework for SCFAs as key effectors of the gut–eye axis. We examine experimental, metabolomic, and clinical data linking SCFA signaling to ocular diseases and identify major challenges to developing therapies. By clarifying these mechanisms and constraints, the review offers a roadmap for advancing microbiome‐based precision treatments in ophthalmology. It aims to provide a comprehensive overview of SCFAs and their structural and functional relationships with eye health. Topics include SCFA biosynthesis, the mechanisms affecting ocular health, and a detailed review of evidence on disease links and therapeutic prospects. The focus is on how changes in the gut microbiota and in SCFA production may relate to the onset of ocular disease. Additionally, the review highlights gaps in understanding how SCFAs influence the gut–eye axis, promoting clearer insight into their therapeutic potential. By analyzing both the benefits and challenges of SCFAs, this work seeks to open new research directions and interventions to improve eye health through diet or microbiome‐targeted approaches.

## 2 SCFA Biosynthesis and Systemic Distribution

### 2.1 Microbial Fermentation Pathways

**FIGURE 1:**
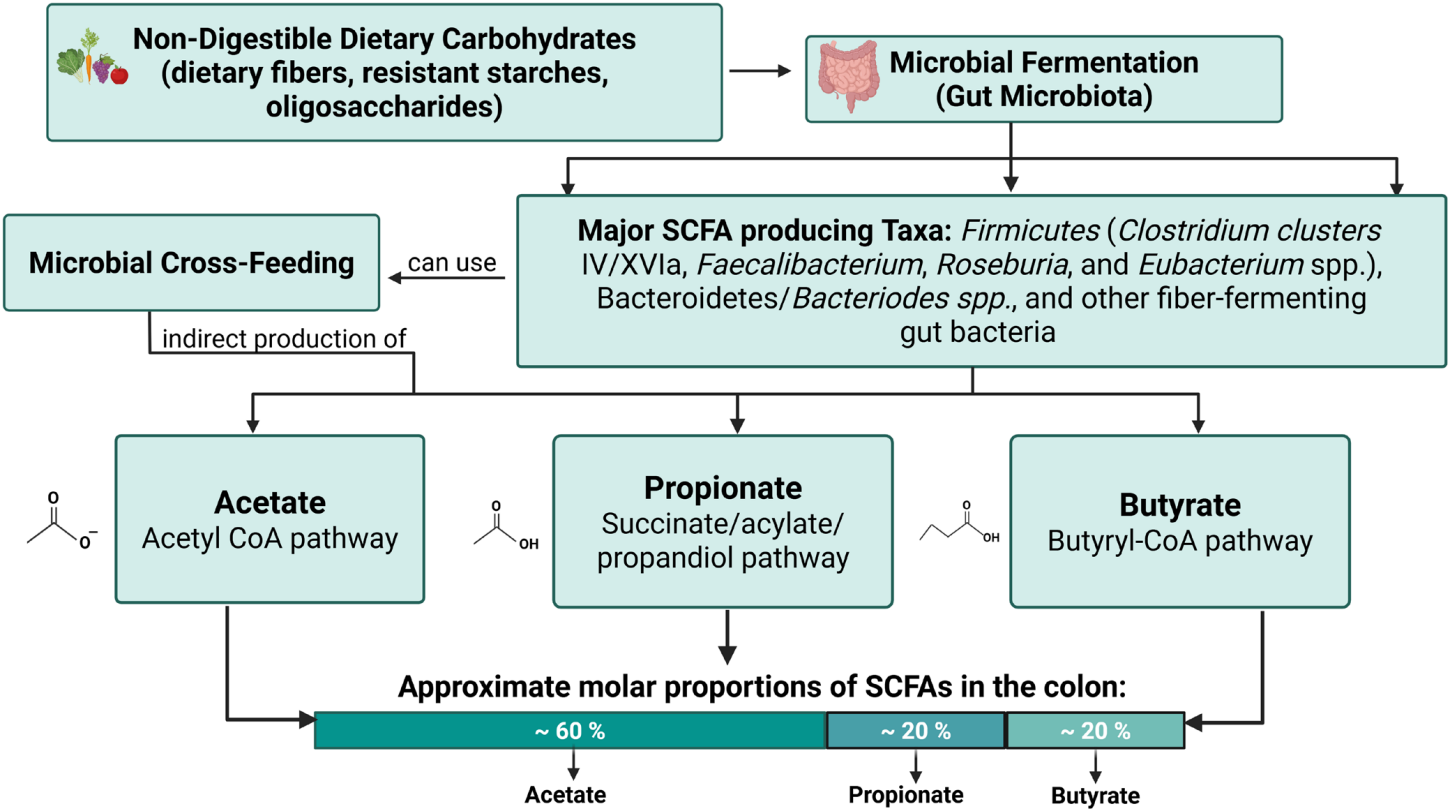
SCFAs are key modulators of intestinal homeostasis. SCFAs are produced from microbial fermentation of dietary fibers. The three most common SCFAs found within the human body are acetate, propionate, and butyrate. Primary SCFA‐producing taxa are *Bioteriodetes*, *Firmicutes*, *Clostridium clusters* IV/XIVa. Biochemical pathways that contribute to SCFA production are the acetyl‐CoA, succinate, acrylate, butyryl‐CoA, and acetate CoA‐transferase pathways. The illustration was created using BioRender.com

SCFA production begins with the fermentation of complex dietary fibers that are not digestible by human enzymes (Mukhopadhya and Louis 2025). These fibers serve as vital substrates that fuel the diverse microbial population residing in the gut (Kumar et al. 2020). Microbial fermentation, a process in which the gut microbiota converts these fibers into SCFAs, is a key mechanism by which dietary components affect health and disease (He et al. 2020). Key butyrate‐producing genera include *Faecalibacterium*, *Roseburia*, and *Eubacterium* (Facchin et al. 2024; Tsukuda et al. 2021). These microbial species play major roles in butyrate production through direct fermentation and metabolic cross‐feeding interactions within the gut microbial community, utilizing substrates and intermediate metabolites generated during fiber fermentation (Teichmann and Cockburn 2021; Kim et al. 2013). Their ability to thrive in diverse gut environments underscores the importance of a balanced and varied diet rich in fiber (Kumar et al. 2025; Annunziata et al. 2020). Different fibers favor the production of distinct SCFAs (Figure 1). For instance, pectin, often found in fruits and vegetables, predominantly supports the growth of bacteria that produce acetate (Bang et al. 2018). At the same time, inulin and other fructans tend to favor propionate‐producing genera such as *Bacteroides* and *Prevotella* (Beukema et al. 2020). The selective fermentation of these fibers by specific microbial species not only broadens the range of SCFAs produced but also influences the composition and function of the gut microbial ecosystem (Annunziata et al. 2020; Stuivenberg et al. 2022) (Figure 1).

Microbial cross‐feeding is an important ecological process in the gut microbiome that substantially contributes to SCFA production. Many SCFA‐producing taxa, particularly butyrate producers, rely on metabolic interactions in which complex carbohydrates are initially degraded by primary fermenters such as *Bifidobacterium adolescentis* and *Ruminococcus bromii* , generating intermediate metabolites that are subsequently utilized by secondary fermenters, including members of the *Firmicutes* and *Bacteroidetes* phyla (Kim et al. 2024; Rios‐Covian et al. 2016). This process is particularly important because several key SCFA‐producing taxa rely, at least in part, on intermediates generated by primary degraders rather than exclusively fermenting complex dietary fibers themselves. In vitro studies have shown that *Bifidobacterium*‐derived acetate and lactate can be utilized by *Roseburia*, *Eubacterium*, and *Anaerobutyricum* species to produce butyrate (Tsukuda et al. 2021; Rios‐Covian et al. 2016). Similarly, lactate can be converted to propionate by *Veillonella* and *Propionibacterium* species (Rios‐Covian et al. 2016). Acetate production may also occur via interspecies substrate exchange involving formate in the Wood‐Ljungdahl pathway (reductive acetyl‐CoA) (Kim et al. 2024). These cooperative metabolic interactions highlight that SCFA production reflects not only the abundance of individual microbial taxa but also the functional organization of the broader microbial community.

Although acetate, propionate, and butyrate are frequently grouped together as SCFAs, they differ substantially in abundance, metabolism, receptor preference, and systemic distribution. Acetate is generally the most abundant circulating SCFA, propionate serves important metabolic functions in the liver, and butyrate exhibits particularly potent immunoregulatory and epigenetic effects despite lower systemic concentrations (He et al. 2020; Mukhopadhya and Louis 2025). These distinctions are important when interpreting studies investigating SCFA‐mediated effects in extra‐intestinal tissues.

Human milk is the primary source of nutrition for infants, contributing to age‐appropriate development and growth, shaping the infant gut microbiome, and providing protection against infections (Duman et al. 2024; Lewis et al. 2017). Human milk is composed of water and other bioactive components, such as human milk oligosaccharides (HMOs) (Duman et al. 2024). HMOs are the crucial solid components of human milk that contribute to the development of the infant gut microbiome (Duman et al. 2024). The infant gut microbiome is unable to digest HMOs directly; thus, the nutritional benefits that HMOs provide are derived via fermentation by *Bifidobacterium*, which promotes the production of SCFAs, namely butyrate, propionate, and acetate (Duman et al. 2024; De Bruyn et al. 2024) (Table 1). This SCFA production helps maintain the pH of the infant gut microbiome and boosts immunity against potential infections (Duman et al. 2024). Studies have shown that the incorporation of HMOs, alone and in combination with pre‐ and probiotics, reduces inflammation, thereby decreasing the risk of gastrointestinal and metabolic disorders (De Bruyn et al. 2024). Furthermore, HMOs are safe additions to infant formulas, which could be an efficient route to addressing the nutritional deficiencies and reduced immunity common in preterm infants (Lewis et al. 2017). This would, in turn, reduce the risk of preterm infants developing immune‐related diseases and diseases of prematurity, including ROP (Lewis et al. 2017).

**TABLE 1:** SCFA biosynthesis, major microbial producers, and dietary substrates.

SCFA	Primary microbial producers	Key fermentation pathways	Preferred dietary substrates	Relative abundance/systemic relevance
Acetate	*Bacteroides* spp., *Bifidobacterium* spp., *Akkermansia muciniphila and* several *Firmicutes*	Acetyl‐CoA pathway (dominant) Wood‐Ljungdahl pathway in acetogenic bacteria (minor contributor in the)	Resistant starch, soluble fibers, pectin, inulin and simple oligosaccharides	Most abundant SCFA in gut and circulation Readily enters systemic circulation Serves as substrate for cholesterol and lipid synthesis Precursor for butyrate production
Propionate	*Bacteroides* spp., *Veillonella* spp., *Prevotella* spp., *Phascolarctobacterium spp* (succinate‐utilizing propionate producers).	Succinate pathway (dominant) Acrylate pathway Propanediol pathway	Pectin, inulin, hemicellulose, arabinoxylans and fucose/rhamnose	Moderate abundance Preferential hepatic uptake Major substrate for hepatic gluconeognesis Regulates lipid metabolism, satiety signaling and immune response via GPR41/43
Butyrate	*Faecalibacterium prausnitzii* , *Roseburia* spp., *Eubacterium rectale* , *Anaerobutyricum* spp.	Acetyl‐CoA → butyryl‐CoA → butyrate (mostly via butyryl‐CoA: acetate CoA‐transferase and less commonly via butyrate kinase)	Resistant starch, inulin, β‐glucans hemicellulose and acetate/lactate generated through microbial cross‐feeding.	Lower luminal abundance but **high biological potency** Primary energy source for colonocytes Strong anti‐inflammatory and epigenetic regulator Emerging evidence supports roles in retinal immune regulation, microglial homeostasis, blood‐retinal barrier integrity, and protection against retinal degeneration

Abbreviations: Acetyl‐CoA, acetyl coenzyme A; butyryl‐CoA, butyryl coenzyme A; CoA, coenzyme A; GPR41, G protein–coupled receptor 41; GPR43, G protein–coupled receptor 43; SCFA, short‐chain fatty acid; spp., species; β‐glucans, beta‐glucans.

Moreover, the systemic distribution of these SCFAs is of paramount importance. Once produced, SCFAs are rapidly absorbed into the bloodstream, allowing them to exert systemic effects, including anti‐inflammatory properties and regulation of glucose metabolism (Xie and Lin 2025; Sanna et al. 2019). This showcases the intricate interplay among dietary fiber, microbial fermentation pathways, and the resulting SCFA biosynthesis, reinforcing the importance of maintaining a healthy gut microbiota through a fiber‐rich diet. By understanding these dynamics, we can better appreciate the potential therapeutic roles of SCFAs in health and disease management.

### 2.2 Absorption and Transport

**FIGURE 2:**
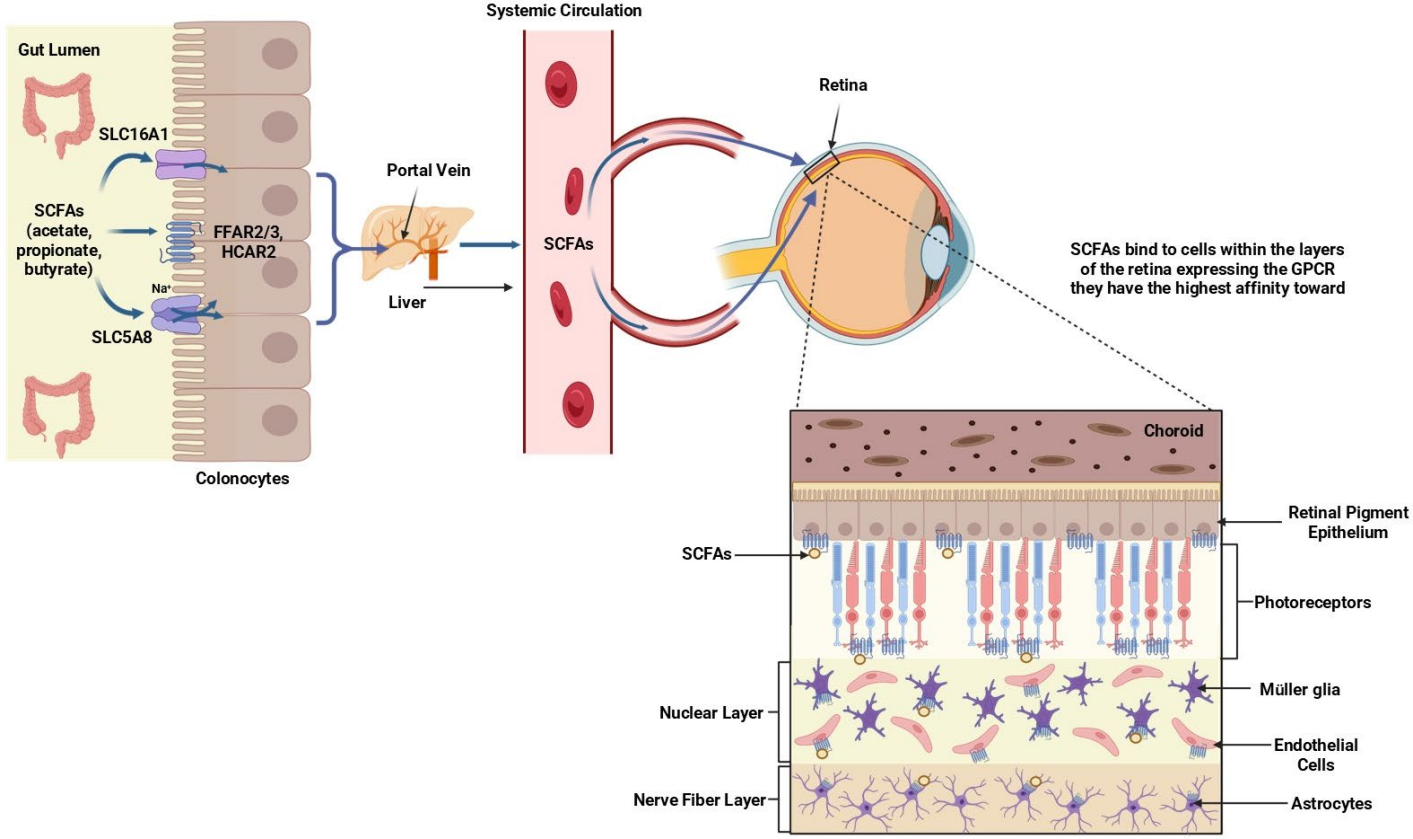
Gut‐retina axis mediating ocular exposure to short‐chain fatty acids. Gut microbiota–derived short‐chain fatty acids (SCFAs) are absorbed by colonic epithelial transporters (SLC16A1 and SLC5A8), enter systemic circulation, and reach the eye. Experimental evidence suggests that circulating SCFAs may reach retinal tissues through systemic circulation and interact with FFAR2/3 and HCAR2 signaling pathways expressed in several ocular cell types. The illustration was created using BioRender.com

Once produced, SCFAs are absorbed by the epithelial cells of the colon, known as colonocytes, via specialized transport mechanisms. The process is primarily facilitated by a range of transporters, including monocarboxylate transporter 1 (MCT1; encoded by SLC16A1), which is coupled to H^+^ transport, and the sodium‐coupled monocarboxylate transporter 1 (SMCT1; encoded by SLC5A8) (Carretta et al. 2021; Miyauchi et al. 2004) (Table 2). These transporters are essential for the effective uptake of SCFAs, specifically, acetate, propionate, and butyrate, each of which is absorbed into the bloodstream and transported along systemic circulation (Miyauchi et al. 2004; Langfeld et al. 2021) (Figure 2).

**TABLE 2:** SCFA transporters and receptors in ocular and systemic tissues.

Transporter/Receptor	Tissue/Ocular expression	Main ligands	Downstream signaling	Functional implications
MCT1 (SLC16A1)	RPE, retinal endothelial cells, Müller glia, photoreceptors; systemic epithelium	Lactate (primary physiological substrate), butyrate and acetate (transported monocarboxylates)	Proton‐coupled transport; metabolic substrate delivery	Metabolic support of retinal cells; coupling of glial–neuronal metabolism; facilitates butyrate entry into ocular tissues
SMCT1 (SLC5A8)	RPE, retinal neurons, expression in retinal immune cells remains under investigation; intestinal epithelium	Butyrate, propionate and acetate (high affinity)	Na^+^‐coupled transport; intracellular HDAC inhibition	Anti‐inflammatory signaling; epigenetic regulation; neuroprotective effects via intracellular SCFA uptake and HDAC inhibition
FFAR2 (GPR43)	Immune cells including macrophages and microglia; possible retinal expression, endothelial cells (systemic)	Acetate, propionate > butyrate	Gαi/Gαq → ↓ cAMP, Ca^2+^ signaling; MAPK modulation	Immune cell regulation; suppression of inflammatory cytokines; modulation of angiogenic responses
FFAR3 (GPR41)	Neurons, vascular endothelial cells, and glial cells: ocular expression remains incompletely characterized	Propionate and butyrate	Gαi → ↓ cAMP; ERK signaling	Metabolic and vascular regulation; indirect modulation of retinal blood flow and inflammation
HCAR2 (GPR109A)	Retinal microglia, RPE, retinal endothelial cells; macrophages	Butyrate, β‐hydroxybutyrate and niacin	Gαi → ↓ NF‐κB; ↑ Nrf2; inflammasome suppression	Potent anti‐inflammatory signaling; suppression of microglial activation; protection against pathological angiogenesis and oxidative stress
Olfr78 (mouse)/OR51E2 (human)	Vascular smooth muscle, endothelial cells	Acetate, propionate	cAMP signaling	Regulation of vascular tone and blood flow

Abbreviations: Ca^2+^, calcium ion; cAMP, cyclic adenosine monophosphate; ERK, extracellular signal‐regulated kinase; FFAR2, free fatty acid receptor 2; FFAR3, free fatty acid receptor 3; GPR109A, G protein–coupled receptor 109A; GPR41, G protein–coupled receptor 41; GPR43, G protein–coupled receptor 43; HCAR2, hydroxycarboxylic acid receptor 2; HDAC, histone deacetylase; MAPK, mitogen‐activated protein kinase; MCT1, monocarboxylate transporter 1; Na^+^, sodium ion; NF‐κB, nuclear factor kappa‐light‐chain‐enhancer of activated B cells; Nrf2, nuclear factor erythroid 2–related factor 2; Olfr78, olfactory receptor 78; OR51E2, olfactory receptor family 51 subfamily E member 2; RPE, retinal pigment epithelium; SCFA, short‐chain fatty acid; SLC16A1, solute carrier family 16 member 1; SLC5A8, solute carrier family 5 member 8; SMCT1, sodium‐coupled monocarboxylate transporter 1.

Upon absorption, SCFAs enter the portal circulation, where they navigate through the hepatic portal vein before reaching the liver (Carretta et al. 2021). SCFA concentrations vary substantially across biological compartments. Within the colonic lumen, total SCFA concentrations typically range from 50 to 150 mM, with acetate accounting for 60%–70%, propionate 15%–25%, and butyrate 10%–20% of the total (Mukhopadhya and Louis 2025; Rios‐Covian et al. 2016). Due to extensive utilization by colonocytes and hepatic first‐pass metabolism, circulating concentrations are markedly lower, generally in the low‐micromolar range. Among circulating SCFAs, acetate is the most abundant, often reaching plasma concentrations of 50–200 μM, whereas propionate and butyrate are typically present at substantially lower concentrations, generally < 10 μM under physiological conditions (Mukhopadhya and Louis 2025; Rios‐Covian et al. 2016). These differences in systemic availability may contribute to distinct biological effects among individual SCFAs and are particularly relevant when considering potential exposure of ocular tissues.

In the liver, SCFAs undergo several critical metabolic processes. The liver is a major site for the conversion and utilization of SCFAs, which participate in various biochemical pathways that support energy metabolism and lipid synthesis (He et al. 2020; Tazoe et al. 2008). For instance, propionate is often converted into glucose via gluconeogenesis, whereas butyrate serves as an important energy source for colonocytes (Tazoe et al. 2008).

The proportion of SCFAs that ultimately reach systemic circulation varies. Still, studies indicate that approximately 60%–80% of the SCFAs produced via microbial fermentation in the gut are absorbed and enter the bloodstream (Mukhopadhya and Louis 2025). This highlights the role of microbial species in our gut microbiota, which ferment dietary fibers and undigested carbohydrates, thereby generating SCFAs that affect not only local gastrointestinal health but also exert systemic effects throughout the body (Mukhopadhya and Louis 2025).

Importantly, individual SCFAs exhibit distinct pharmacokinetic profiles. Acetate readily enters systemic circulation and achieves the highest peripheral concentrations, whereas propionate undergoes substantial hepatic extraction and is closely linked to gluconeogenic pathways. In contrast, butyrate is extensively utilized by colonocytes and generally reaches the circulation at relatively low concentrations despite its potent biological activity (Mukhopadhya and Louis 2025; Rios‐Covian et al. 2016). Consequently, systemic and ocular exposure may differ considerably among SCFA species.

### 2.3 Receptor Biology and Extra‐Intestinal Signaling

SCFAs exert their biological effects through activation of GPCRs, primarily FFAR2/GPR43, FFAR3/GPR41, and HCAR2/GPR109A, as well as through receptor‐independent mechanisms such as HDAC inhibition (He et al. 2020; Carretta et al. 2021). These receptors are expressed on a wide range of cell types, including colonocytes, immune cells, neurons, adipocytes, pancreatic cells, endothelial cells, epithelial cells, and microglia, enabling SCFAs to regulate metabolic, immunological, and neuroinflammatory processes both locally and systemically (He et al. 2020; Carretta et al. 2021; Tazoe et al. 2008). FFAR2 and FFAR3 primarily signal through Gαi/o and Gαq pathways, thereby modulating intracellular cyclic AMP (cAMP), calcium flux, mitogen‐activated protein kinase (MAPK), and extracellular signal‐regulated kinase (ERK) signaling cascades (Carretta et al. 2021; Tazoe et al. 2008). HCAR2 can be activated by butyrate, as well as by endogenous ligands such as β‐hydroxybutyrate and niacin, and is recognized as an important mediator of anti‐inflammatory signaling in immune and epithelial cells (Carretta et al. 2021; Kalkan et al. 2025).

In adipocytes, activation of FFAR2/3 and HCAR2 generally results in coupling to Gαi/o proteins and suppression of adenylyl cyclase activity, leading to reduced cAMP production and downstream modulation of ERK1/2 signaling (Carretta et al. 2021; Tazoe et al. 2008) (Figure 2). In contrast, FFAR2 activation in neutrophils can induce Gαq‐mediated calcium mobilization and reactive oxygen species (ROS) generation, contributing to antimicrobial responses and context‐dependent immune signaling (Carretta et al. 2021). Collectively, SCFA receptor signaling plays a critical role in regulating inflammation, barrier function, metabolism, and vascular homeostasis.

#### 2.3.1 Acetate‐Mediated Signaling

Acetate preferentially activates FFAR2 (GPR43), triggering downstream pathways involved in immune regulation, metabolism, and vascular homeostasis (Kim et al. 2013; Carretta et al. 2021; Tazoe et al. 2008). Recent studies have shown that acetate‐mediated activation of FFAR2 can stimulate AMP‐activated protein kinase (AMPK) signaling, leading to reduced oxidative stress, attenuation of inflammatory cytokine production, and improved vascular integrity (Wada et al. 2025). Additionally, acetate‐FFAR2 signaling has been associated with improved insulin sensitivity and reduced insulin resistance in experimental models of metabolic disease (Wada et al. 2025; Lee et al. 2024). Acetate has also been shown to enhance innate immune responses through increased production of interleukin‐22 (IL‐22), a cytokine involved in epithelial barrier maintenance and mucosal immunity (Zhang et al. 2023; Lee et al. 2024).

#### 2.3.2 Propionate‐Mediated Signaling

Propionate is a potent ligand for both FFAR2 and FFAR3 and exhibits receptor‐specific effects depending on the cellular context (He et al. 2020; Carretta et al. 2021). Through activation of FFAR3, propionate has been shown to reduce the expression of inflammatory cytokines such as IL‐4, IL‐5, and IL‐17A, thereby contributing to anti‐inflammatory immune responses (He et al. 2020). Experimental studies further suggest that propionate‐mediated FFAR3 activation can increase secretion of peptide YY (PYY) and glucagon‐like peptide‐1 (GLP‐1), improving glucose homeostasis and protecting against diet‐induced obesity (Zhang et al. 2023). Propionate signaling through FFAR2 has also been associated with neuroprotective effects in experimental models of Parkinson's disease, highlighting broader roles in gut‐neural communication and systemic immune regulation (Zhang et al. 2023).

#### 2.3.3 Butyrate‐Mediated Signaling

Butyrate exerts the broadest range of biological effects among the SCFAs because it functions both as a GPCR ligand and as a potent HDAC inhibitor (He et al. 2020; Kalkan et al. 2025; Caetano and Castelucci 2022). Butyrate activates FFAR2, FFAR3, and HCAR2/GPR109A, promoting anti‐inflammatory signaling, epithelial barrier integrity, and immune homeostasis (Carretta et al. 2021; Caetano and Castelucci 2022). Activation of HCAR2 and FFAR2 has been shown to enhance mucosal immune function by increasing immunoglobulin A (IgA) secretion and improving epithelial integrity under inflammatory conditions (Kalkan et al. 2025). In addition, butyrate‐mediated FFAR3 signaling reduces expression of pro‐inflammatory mediators including TNF‐α, IL‐6, inducible nitric oxide synthase (iNOS), and monocyte chemoattractant protein‐1 (MCP‐1) (He et al. 2020). Activation of FFAR2/3 by butyrate can also stimulate AMPK signaling and increase the expression of tight junction proteins, thereby strengthening epithelial barrier function (Caetano and Castelucci 2022) (Table 2).

Importantly, many biological effects of butyrate extend beyond receptor‐mediated signaling. Through HDAC inhibition, butyrate regulates gene expression, immune cell differentiation, oxidative stress responses, and cellular metabolism, providing an additional epigenetic mechanism that distinguishes it from acetate and propionate (He et al. 2020; Kalkan et al. 2025; Caetano and Castelucci 2022).

### 2.4 Systemic Distribution and Ocular Exposure

Despite ocular barriers, SCFAs may influence the retina by traversing the blood‐retinal barrier (BRB), a highly selective permeability barrier critical for maintaining retinal homeostasis. Emerging evidence suggests that certain SCFAs can access the retina, likely via transporter‐mediated mechanisms, as monocarboxylate transporters such as SMCT1, SMCT2, and MCT1 are expressed in retinal cells and facilitate the movement of SCFA‐related metabolites across retinal tissues (Martin et al. 2007). However, direct measurements of individual SCFA concentrations within ocular tissues remain extremely limited. As a result, most current assumptions regarding retinal exposure are based on circulating metabolite levels, transporter expression, and biological responses observed after systemic SCFA administration, rather than on direct quantification within the retina itself. In addition, systemic SCFA administration has been associated with modulation of retinal inflammation and vascular pathology in preclinical models (Martin et al. 2007; Qin et al. 2025). GPCRs to which SCFAs can bind are found in various retinal cells, including retinal ganglion cells and Müller glia, where they play critical roles in cellular signaling and response mechanisms (Gambhir et al. 2012; Abdelrahman et al. 2022; Lester et al. 2026) (Figure 2). The activation of these receptors by SCFAs could potentially lead to a cascade of neuroprotective effects, inflammatory responses, and metabolic regulation within the retinal environment (Liu et al. 2018; Nastasi et al. 2017; Schaefer et al. 2022). Therefore, understanding the dynamics of SCFA biosynthesis and their systemic distribution is essential to unraveling their potential role in ocular health. By exploring the interactions among microbial species, SCFA production, and ocular barrier mechanisms, we can gain deeper insights into novel therapeutic strategies to prevent or alleviate ocular diseases, thereby highlighting the invaluable contributions of the gut‐microbiome‐ocular axis.

## 3 Gut–Eye Axis: Mechanistic Insights

### 3.1 Immune Modulation

**FIGURE 3:**
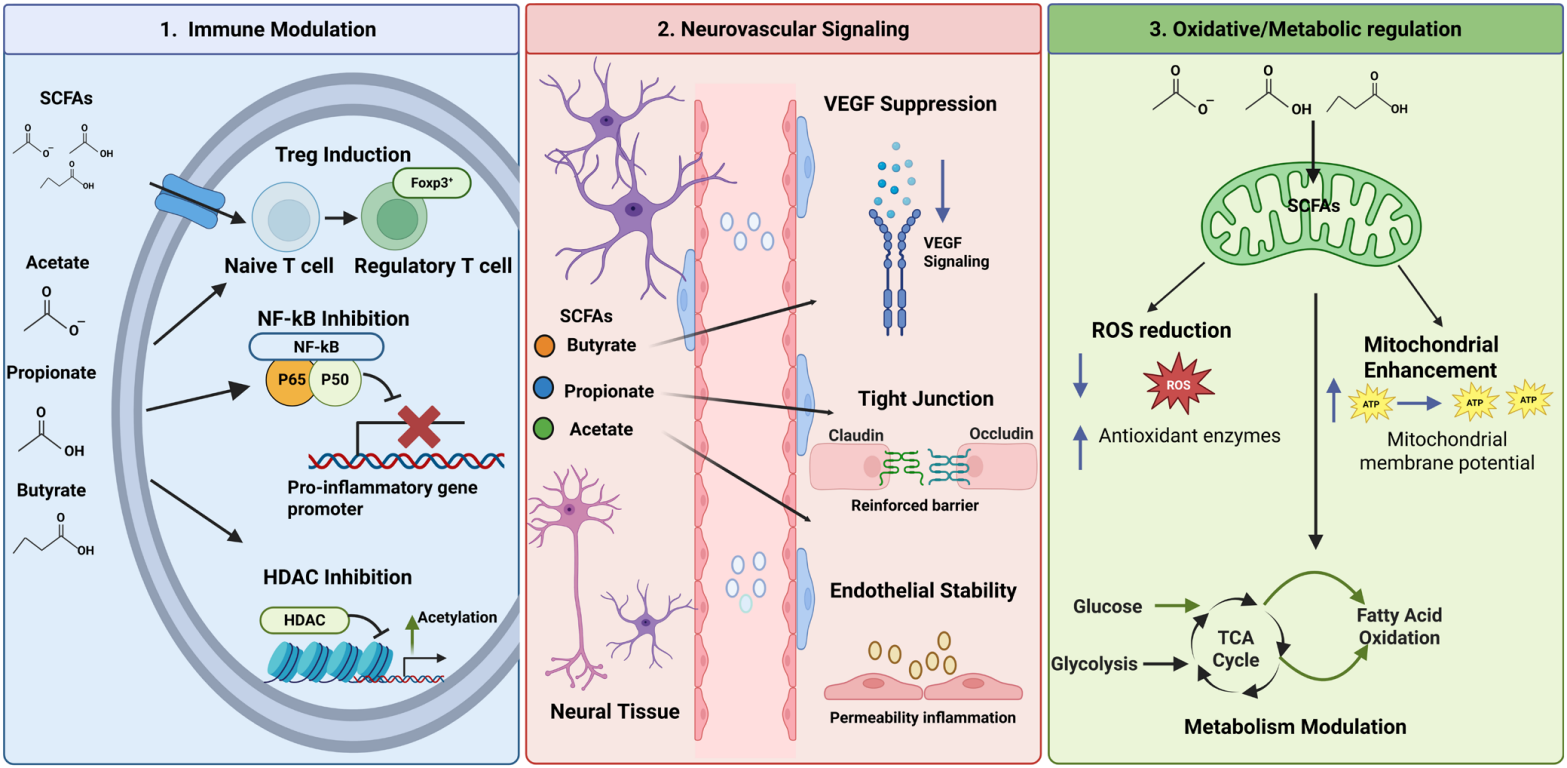
Mechanistic roles of short‐chain fatty acids (SCFAs) in the gut–eye axis. Schematic representation of key mechanisms through which gut microbiota–derived short–chain fatty acids (SCFAs) influence ocular physiology and pathology. (1) Immune modulation: SCFAs promote regulatory T cell (Treg) differentiation, suppress nuclear factor‐κB (NF‐κB) signaling, and inhibit histone deacetylases (HDACs), leading to reduced pro‐inflammatory gene expression. (2) Neurovascular signaling: SCFAs reinforce blood–retinal barrier integrity by enhancing endothelial tight junction proteins (e.g., claudin, occludin), stabilizing endothelial cell function, and modulating angiogenic pathways, including VEGF signaling. (3) Oxidative and metabolic regulation: SCFAs improve mitochondrial function, reduce reactive oxygen species (ROS) accumulation, enhance antioxidant defenses (e.g., superoxide dismutase and catalase), and support metabolic homeostasis through coordinated regulation of glycolysis, the tricarboxylic acid (TCA) cycle, and fatty acid oxidation. Collectively, these mechanisms highlight SCFAs as key mediators linking gut microbial metabolism to retinal immune, vascular, and metabolic function. The illustration was created using BioRender.com

SCFAs regulate immune responses through receptor‐dependent signaling and, particularly for butyrate, by inhibiting HDACs (Mann et al. 2024; He et al. 2020) (Figure 3). In intestinal and peripheral immune systems, these mechanisms have been associated with regulatory T‐cell differentiation, modulation of macrophage activity, and suppression of inflammatory cytokine production (Liu et al. 2018; Singh et al. 2018; Chang et al. 2014). Butyrate and propionate have been most consistently implicated in these immunoregulatory effects, including promotion of regulatory T‐cell responses and increased production of IL‐10 and TGF‐β in experimental systems (Mann et al. 2024; He et al. 2020). Direct evidence that these mechanisms operate within ocular tissues remains limited. Experimental studies in uveitis indicate that SCFA administration can reduce ocular inflammation and alter systemic immune responses (Nakamura et al. 2017; Chen et al. 2021); however, the precise contributions of retinal or other local ocular immune cells have not been fully defined. Therefore, evidence from gastrointestinal and peripheral immune systems should not be assumed to apply directly to the retina or other ocular tissues. Overall, SCFA‐mediated immune regulation is a biologically plausible mechanism within the gut–eye axis, but its role in retinal disease remains incompletely established. Ocular‐specific studies are needed to determine the relevant SCFAs, target cell populations, receptors, and downstream pathways involved.

### 3.2 Vascular Protection

Vascular protection signaling represents a critical interface through which SCFAs may influence retinal homeostasis and vascular integrity. One potential mechanism involves the regulation of vascular endothelial growth factor (VEGF), a central mediator of angiogenesis. Although direct evidence in ocular systems remains limited, studies in vascular and other tissues demonstrate that HDAC activity is closely linked to angiogenic signaling pathways, including regulation of hypoxia‐inducible factor 1α (HIF‐1α) and VEGF expression (Chang et al. 2014; Ellis et al. 2009). SCFAs, particularly butyrate and, to a lesser extent, propionate, can inhibit HDAC activity. Available studies most consistently implicate Class I HDACs, including HDAC1, HDAC2, HDAC3, and HDAC8; however, effects on Class IIa isoforms, including HDAC4, HDAC5, HDAC7, and HDAC9, may vary by experimental system. Butyrate generally exhibits greater HDAC‐inhibitory activity than propionate (Deng et al. 2025). The relative contributions of specific HDAC isoforms to vascular and retinal responses remain incompletely defined. In support of this, HDAC inhibition has been shown to suppress VEGF‐driven angiogenesis and endothelial proliferation (Rössig et al. 2002), suggesting a mechanism by which SCFAs could restrain aberrant neovascularization.

Beyond angiogenesis, SCFAs exert important vascular protective effects by attenuating endothelial inflammation. SCFAs have been shown to suppress endothelial activation induced by inflammatory stimuli such as lipopolysaccharide (LPS) or tumor necrosis factor‐α (TNF‐α), thereby reducing the production of pro‐inflammatory cytokines (e.g., IL‐6, IL‐8) and the expression of adhesion molecules such as VCAM‐ 1 (Li et al. 2018). These anti‐inflammatory actions are mediated through both GPR41/43 signaling and HDAC inhibition, which collectively modulate key inflammatory pathways, including NF‐κB and MAPK signaling (Li et al. 2021). Notably, HDAC‐dependent mechanisms regulate endothelial cytokine production and leukocyte adhesion, processes central to vascular inflammation and retinal pathology.

In addition to anti‐inflammatory effects, SCFAs contribute to endothelial barrier stabilization. Tight junction complexes composed of proteins such as occludin and claudins are essential for maintaining vascular barrier integrity, including that of the BRB. Evidence from endothelial and blood–brain barrier models indicates that SCFAs reduce paracellular permeability and enhance endothelial cohesion, likely through HDAC inhibition–mediated upregulation of junctional proteins (Miyoshi et al. 2008). For example, propionate and butyrate decrease endothelial permeability at concentrations ranging from approximately 0.1–2.0 mM in endothelial cell models, a range considered physiologically relevant for circulating SCFAs following microbial fermentation, without inducing significant cytotoxicity (Miyoshi et al. 2008). At the same time, pharmacologic HDAC inhibitors produce similar barrier‐protective effects, supporting a central role for epigenetic regulation in this process (Miyoshi et al. 2008). Furthermore, butyrate has been shown to strengthen endothelial junctions by increasing the expression of VE‐cadherin and claudin‐5, while simultaneously preserving mitochondrial function and metabolic homeostasis in endothelial cells (Miyoshi et al. 2008).

SCFAs may also influence endothelial function by modulating nitric oxide (NO) signaling and vascular tone. Endothelial nitric oxide synthase (eNOS) is a key regulator of vasorelaxation and angiogenic signaling; however, HDAC inhibition has been shown to suppress eNOS expression and angiogenesis in endothelial cells, suggesting a regulatory role in limiting excessive vascular activity (Rössig et al. 2002).

Collectively, these findings support a model in which SCFAs exert multifaceted vascular protective effects by simultaneously dampening endothelial inflammation, stabilizing barrier integrity, and modulating angiogenic signaling through HDAC‐dependent mechanisms. While these effects are well established in peripheral and central vascular systems, their direct relevance to retinal microvasculature and BRB function remains to be fully elucidated. Nonetheless, these mechanisms provide biological plausibility for a role of SCFAs in neurovascular regulation. However, direct evidence demonstrating that these pathways operate in retinal tissues remains limited, and their relevance to human retinal disease requires further investigation (Figure 3).

### 3.3 Oxidative Stress & Metabolic Regulation

SCFAs, especially butyrate, play a crucial role in cellular metabolism and redox balance. Butyrate acts as a metabolic substrate, fueling mitochondrial oxidative pathways and supporting respiration and energy production across various cell types. In vitro studies using pancreatic β‐cells, neuronal cultures, and other metabolically active cell systems have demonstrated that acetate and butyrate improve mitochondrial function under oxidative stress by enhancing oxidative phosphorylation and preserving mitochondrial dynamics, including fusion and fission. These bioenergetic effects are particularly important in metabolically active tissues where mitochondrial integrity is vital for cellular health (Zhang et al. 2021; Hu et al. 2020). Much of the current evidence supporting these mechanisms derives from in vitro cellular studies and preclinical animal models, whereas direct evidence in retinal tissues remains comparatively limited. Consequently, many mechanistic links between SCFA signaling, oxidative stress regulation, and retinal protection are inferred from studies performed in non‐ocular tissues and subsequently evaluated in ocular disease models.

Beyond their metabolic functions, SCFAs also influence the regulation of oxidative stress. Both acetate and butyrate reduce reactive oxygen species (ROS) buildup and prevent mitochondrial dysfunction under metabolic and oxidative stress conditions. Butyrate, in particular, enhances mitochondrial function and shields neurons from oxidative damage by restoring mitochondrial quality‐control mechanisms such as mitophagy. These findings underscore that SCFAs directly support mitochondrial health, which largely determines intracellular ROS levels (Hu et al. 2020; Cho et al. 2024).

Mechanistically, SCFAs maintain redox balance through metabolic and signaling pathways. Experimental studies conducted primarily in cellular and animal models have shown that SCFAs can modulate redox‐sensitive signaling pathways, including activation of the Kelch‐like ECH‐associated protein 1 (Keap1) and nuclear factor erythroid 2–related factor 2 (Nrf2) pathway. Nrf2 activation increases the expression of antioxidant enzymes, aiding in ROS detoxification and redox homeostasis, thereby protecting tissues from oxidative damage. Additionally, SCFAs act as HDAC inhibitors, providing an epigenetic mechanism to regulate genes involved in oxidative stress responses and mitochondrial function (González‐Bosch et al. 2021; Ngo and Duennwald 2022).

SCFAs also influence broader metabolic regulation by promoting mitochondrial biogenesis and cellular energy production. Their signaling can activate pathways like AMPK (AMP‐activated protein kinase) and PGC‐1α (Peroxisome proliferator‐activated receptor gamma coactivator 1‐alpha), which are crucial for mitochondrial biogenesis and metabolic adaptation. Through these pathways, SCFAs enhance metabolic flexibility and bolster cells' capacity to handle energetic and oxidative stresses (Réka et al. 2025).

These mechanisms are particularly relevant to retinal health, given the established role of oxidative stress in neurovascular degeneration; however, direct validation of several of these pathways in retinal tissues remains limited (Wang et al. 2022; Rohowetz et al. 2018). By improving mitochondrial function, reducing ROS buildup, and activating antioxidant pathways, SCFAs may help preserve cellular integrity and protect against retinal stress injuries.

In summary, the gut‐eye axis is a crucial interface in which microbial metabolites, such as SCFAs, influence multiple aspects of ocular health, including immune modulation, neurovascular signaling, and oxidative stress management (Campagnoli et al. 2023; Tsang et al. 2019). These findings underscore the significant interplay among the gut microbiota, their metabolic byproducts, and retinal health, paving the way for potential therapeutic strategies to enhance ocular function through dietary interventions and microbiome modulation (Oxenrider et al. 2025; Kim et al. 2019; Duysburgh et al. 2025). Taken together, current evidence supporting SCFA‐mediated regulation of oxidative stress consists largely of mechanistic studies and preclinical disease models. While these findings provide biological plausibility for retinal protection, additional retinal‐specific and clinical studies will be required to establish the relevance of these mechanisms in human ocular disease.

### 3.4 Role of SCFA‐Producing Microbiome in Retinal Diseases

The composition and metabolic activity of the gut microbiome have been increasingly linked to retinal health and disease through the gut‐eye axis. While several microbial taxa, including *Faecalibacterium*, *Roseburia*, *Bacteroides*, and other SCFA‐producing organisms, have been implicated in retinal disorders, emerging evidence suggests that functional metabolic output, particularly SCFA production capacity, may be more informative than the abundance of individual bacterial taxa alone (Facchin et al. 2024; Nguyen et al. 2024). Consequently, current investigations are increasingly focused on identifying disease‐associated alterations in microbial metabolic pathways rather than defining specific microbial signatures.

Microbiome studies of retinal diseases have revealed recurring patterns of dysbiosis characterized by reduced abundance of SCFA‐producing taxa and alterations in pathways involved in carbohydrate fermentation and microbial metabolite production (Nguyen et al. 2024). These observations are discussed in greater detail below, including current limitations related to study design, reproducibility, and functional interpretation of microbiome data.

Importantly, changes in microbial composition do not necessarily translate into altered metabolite production. Because SCFA biosynthesis depends on complex microbial interactions, including cross‐feeding among multiple bacterial species, taxonomic profiling alone may provide an incomplete assessment of functional microbial activity. Integration of microbiome sequencing with metabolomic analyses has therefore emerged as an important strategy for evaluating whether alterations in SCFA production contribute to retinal disease‐associated inflammatory, vascular, or neurodegenerative processes.

Collectively, available evidence supports an association between microbiome dysbiosis, altered SCFA‐related metabolic pathways, and retinal disease; however, direct causal relationships remain incompletely established. Future longitudinal and multi‐omics studies incorporating microbial composition, metabolite quantification, and disease phenotyping will be necessary to determine the functional significance of SCFA‐producing microbial communities in retinal pathology.

#### 3.4.1 Dysbiosis Patterns in Retinal Disease

Dysbiosis, defined as an alteration in gut microbial composition and diversity, has been increasingly associated with a range of retinal diseases, including DR, AMD, and retinopathy of prematurity (ROP) (Tirziu et al. 2024). Clinical and preclinical studies consistently demonstrate disease‐specific microbial signatures across these conditions. Despite increasing interest in microbiome‐associated retinal disease, the reliability and reproducibility of reported microbial signatures require careful consideration. Many studies are based on relatively small cohorts and utilize different sequencing platforms, analytical pipelines, and taxonomic classification methods, which can influence the microbial taxa identified. Furthermore, patient populations often differ substantially with respect to age, geographic location, dietary habits, medication use, metabolic status, and disease severity, all factors known to affect microbiome composition independently of retinal disease. Consequently, while recurrent patterns of dysbiosis have been reported across several retinal disorders, the specific taxa associated with disease are not always consistent among studies. In DR, reduced abundance of beneficial commensal bacteria, particularly those associated with anti‐inflammatory functions, is accompanied by increased systemic inflammation and oxidative stress, thereby contributing to retinal vascular damage and disease progression (Sanna et al. 2019; Jabbehdari and Sallam 2022; Kim et al. 2023). Similarly, AMD has been associated with shifts in gut microbial composition characterized by enrichment of pro‐inflammatory taxa and depletion of beneficial microbes, suggesting a role for microbiota‐driven immune dysregulation in retinal degeneration (Rowan et al. 2017; Napolitano et al. 2021). In the context of ROP, emerging evidence points to reduced microbial diversity and altered early‐life microbial colonization patterns, which may influence retinal vascular development and disease susceptibility (Stuivenberg et al. 2022; Nguyen et al. 2024; Win et al. 2021).

While these studies provide strong evidence linking gut dysbiosis to retinal pathology, the functional metabolic consequences of these microbial alterations are only beginning to be understood. Notably, several of the taxa reported to be reduced in these conditions are known contributors to SCFA production. This raises the possibility that dysbiosis‐associated loss of SCFA‐producing bacteria could reduce systemic SCFA availability. Given the established roles of SCFAs in regulating inflammation, oxidative stress, and vascular homeostasis, these changes may represent a key mechanistic link between gut microbiome alterations and retinal disease pathogenesis.

Collectively, these findings support a working model in which microbiome dysbiosis contributes to retinal disease not only through compositional changes but also via disruption of microbial metabolic outputs, particularly SCFA production. However, direct causal evidence linking altered SCFA levels to retinal pathology remains limited, underscoring the need for further studies to define these relationships and to evaluate whether restoration of SCFA‐producing microbial communities can serve as a therapeutic strategy within the gut–eye axis framework.

Another important limitation is that most available studies are cross‐sectional in design and therefore cannot determine whether microbiome alterations contribute to disease pathogenesis or arise as a consequence of disease‐associated physiological and metabolic changes. In addition, many investigations rely on taxonomic profiling alone, without direct measurement of microbial metabolites such as SCFAs. As a result, inferred functional changes in SCFA production often remain indirect. Longitudinal, multi‐center studies integrating microbiome, metabolomic, and clinical data will be necessary to improve confidence in reported associations and establish mechanistic relevance.

#### 3.4.2 Host Interfaces

Understanding the interfaces between the gut microbiome and the host is essential for elucidating the mechanisms linking microbial metabolism to the pathophysiology of retinal disease. The intestinal epithelial barrier serves as the first critical interface, functioning as a selective, tightly regulated barrier that prevents the translocation of harmful pathogens while permitting the absorption of nutrients and beneficial microbial metabolites, including SCFAs (Campagnoli et al. 2023; Tirziu et al. 2024; Zysset‐Burri et al. 2023; Scuderi et al. 2021). Maintenance of gut barrier integrity is therefore crucial for systemic homeostasis; disruption of this barrier can lead to increased permeability (“leaky gut”), facilitating the entry of microbial products into circulation and promoting systemic inflammation, a key contributor to retinal vascular and neurodegenerative changes (Campagnoli et al. 2023; Tirziu et al. 2024; Zysset‐Burri et al. 2023; Scuderi et al. 2021).

Following absorption, SCFAs and other microbial metabolites are distributed via the circulatory system to distal organs, where they influence immune, metabolic, and vascular processes. Although direct evidence of SCFA transport across the BRB remains limited, their systemic immunomodulatory and metabolic effects suggest an indirect influence on retinal health. The BRB itself constitutes a second critical interface, maintaining retinal homeostasis by tightly regulating molecular exchange and controlling immune cell infiltration into the retinal microenvironment (Xie and Lin 2025; Nguyen et al. 2024; Scuderi et al. 2021). This highly specialized barrier, composed of endothelial cells, pericytes, and supporting glial elements, is essential for preserving immune privilege and preventing excessive inflammatory responses within the retina.

Disruption at either of these interfaces—the gut barrier or the BRB—may have significant consequences for retinal integrity. Compromise of gut barrier function can amplify systemic inflammatory signaling, while dysfunction of the BRB can facilitate leukocyte infiltration, vascular leakage, and tissue damage. Together, these interconnected interfaces form a bidirectional axis through which gut‐derived metabolites and inflammatory mediators may influence retinal physiology and pathology. Understanding these crosstalk mechanisms provides a critical framework for exploring therapeutic strategies to restore barrier integrity and modulate microbiome‐derived metabolic signaling in retinal diseases.

#### 3.4.3 Confounders and Methods

Consideration of key confounding factors is essential for accurately interpreting the complex relationship between the gut microbiome and retinal diseases. Among these, dietary composition, particularly fiber intake, is a major determinant of gut microbial diversity and metabolic output, including SCFA production (Mukhopadhya and Louis 2025; Ciurariu et al. 2025). Although dietary patterns are known to influence microbiome composition and SCFA availability, relatively few studies have directly evaluated the relationship between diet, SCFA production, and retinal disease activity. Consequently, diet should currently be viewed primarily as a potential confounding variable in gut‐retina axis studies rather than an established determinant of retinal disease progression. Future studies integrating detailed dietary assessments with microbiome and metabolomic profiling will be necessary to determine whether diet‐dependent changes in SCFA production influence retinal disease susceptibility or progression.

Pharmacological interventions, especially the use of antibiotics, represent another major confounding factor. Antibiotic exposure can significantly disrupt gut microbial communities, leading to depletion of beneficial taxa and reduced SCFA production capacity, with potential downstream effects on systemic inflammation and retinal health (Simmonds et al. 2025; Rassi et al. 2025). Additionally, metabolic status—particularly glycemic control in individuals with diabetes—has been shown to influence gut microbiome composition and function. Fluctuations in blood glucose levels can alter microbial ecology, potentially exacerbating dysbiosis and its associated metabolic consequences (Adhikary et al. 2026; Chong et al. 2024; Takkar et al. 2026).

From a methodological standpoint, advances in high‐throughput sequencing technologies have substantially enhanced our ability to characterize the gut microbiome. Techniques such as 16S ribosomal RNA sequencing allow for taxonomic profiling of microbial communities, while whole‐genome metagenomic approaches provide deeper insights into microbial functional capacity and metabolic potential (Adhikary et al. 2026; Ye et al. 2021). Together, these tools have been instrumental in identifying disease‐associated dysbiosis patterns and in elucidating potential functional links between microbiome alterations and host physiology.

Despite these advances, variability in study design, cohort characteristics, dietary factors, and sequencing methodologies remains a significant challenge in the field. Careful consideration of these confounders is therefore critical when interpreting findings and drawing conclusions about the role of the gut–eye axis in the pathogenesis of retinal disease. Standardization of analytical approaches and integration of multi‐omics data will be essential for advancing our understanding of microbiome‐driven mechanisms and their translational relevance.

## 4 Evidence Linking SCFAs to Ocular Diseases

Building on the biosynthetic, transport, receptor, and mechanistic frameworks described above, this section evaluates the evidence linking SCFAs to ocular disease phenotypes. Rather than organizing the evidence solely by individual diseases, we stratify the literature according to the type and strength of evidence: experimental perturbation studies, direct SCFA supplementation studies, receptor‐ or pathway‐based studies, and human associative or translational data. To facilitate interpretation, the literature discussed in this section is categorized according to level of evidence. Mechanistic and experimental studies are considered separately from animal efficacy studies, observational microbiome and metabolomic investigations, and emerging clinical or translational data. This framework allows differentiation between findings that support causal relationships and those that identify associations requiring further validation.

Despite growing interest in SCFAs as mediators of the gut‐eye axis, the strength of evidence remains uneven across ocular diseases. Experimental studies in uveitis and ocular surface inflammation provide direct evidence that SCFA supplementation can modulate disease severity and inflammatory responses. In contrast, evidence in diabetic retinopathy, age‐related macular degeneration, and glaucoma is derived predominantly from microbiome association studies, metabolomic profiling, or indirect mechanistic inference. These observational studies demonstrate correlations between dysbiosis, altered SCFA‐producing taxa, and disease phenotypes but do not establish causality. Furthermore, substantial heterogeneity exists among studies with respect to patient populations, dietary habits, sequencing methodologies, and analytical pipelines, complicating cross‐study comparisons. Collectively, the available literature supports a biologically plausible role for SCFAs in ocular disease; however, the overall evidence base remains strongest for inflammatory ocular disorders and less definitive for degenerative and vascular retinal diseases.

### 4.1 Experimental Evidence Supporting a Causal Role for SCFAs in Ocular Diseases

Experimental studies provide the strongest support for a causal role of SCFAs in ocular disease by enabling controlled perturbation of the gut microbiome, direct supplementation of metabolites, and targeted interrogation of SCFA‐sensing pathways. These approaches demonstrate that modulation of SCFA availability or signaling can influence ocular inflammation, vascular pathology, and retinal homeostasis within the gut‐eye axis framework.

Microbiome disruption models have been especially informative. In experimental autoimmune uveitis, altering the gut microbiota reduces disease severity, an effect linked to decreased activation and trafficking of autoreactive T cells from the intestine to the eye (Nakamura et al. 2017). Similarly, systemic administration of SCFAs has been shown to suppress ocular inflammation in endotoxin‐induced uveitis, with detectable levels of SCFAs in ocular tissues following treatment, supporting the concept that circulating microbial metabolites can access and act within the eye (Chen et al. 2021). These findings provide functional evidence that microbiome‐derived metabolites are not only associated with but also actively regulate ocular inflammatory responses. In addition to immune modulation, SCFAs play a critical role in maintaining barrier integrity, particularly within the intestinal epithelium. This includes the regulation of epithelial tight junctions and metabolic support of colonocytes, processes that help preserve mucosal homeostasis, and may indirectly influence distal barrier systems such as the blood‐retinal barrier (Kelly et al. 2015; Peng et al. 2009).

Direct supplementation studies further reinforce this relationship. Administration of acetate, propionate, or butyrate attenuates inflammatory responses and improves retinal outcomes across multiple experimental systems (Nakamura et al. 2017; Chen et al. 2021; Huang et al. 2023). Beyond uveitis, oral tributyrin, a butyrate prodrug, reduces ocular surface inflammation in a murine model of desiccating stress, supporting a functional role for gut‐derived butyrate in ocular surface disease (Schaefer et al. 2022). In diabetic contexts, sodium butyrate ameliorates retinal dysfunction and structural damage (Huang et al. 2023).

Receptor‐targeted studies provide additional mechanistic specificity. SCFAs signal through G‐protein‐coupled receptors, including FFAR2 (GPR43), FFAR3 (GPR41), and GPR109A (Kim et al. 2013; Singh et al. 2014). While direct receptor‐based studies in ocular disease remain limited, SCFA‐mediated receptor activation has been shown to regulate inflammatory signaling and barrier integrity in multiple tissues (Maslowski et al. 2009; Smith et al. 2013). In addition to receptor‐mediated signaling, SCFAs exert epigenetic effects through HDAC inhibition, further contributing to immune regulation, barrier integrity, and metabolic homeostasis, as discussed in Section 3.

### 4.2 Evidence Across Ocular Disease Contexts

While experimental studies establish a causal framework, disease‐specific investigations provide insight into how SCFA‐related mechanisms manifest across distinct ocular conditions (Table 3). The strength and nature of evidence vary substantially by disease, with immune‐mediated conditions demonstrating the most direct experimental support and metabolic or degenerative diseases relying more heavily on preclinical or associative data. Together, these disease contexts illustrate how SCFA signaling converges on shared physiological pathways, including immune regulation, metabolic homeostasis, and vascular function, while manifesting in a tissue‐specific manner across distinct ocular compartments. These pathways, in turn, intersect with key retinal cell populations, including microglia, Müller glia, and retinal pigment epithelial cells, which serve as central regulators of inflammatory responses, barrier integrity, and metabolic homeostasis within the retina (Yang et al. 2026; Murenu et al. 2022). Across ocular diseases, SCFA‐related effects converge on several shared mechanisms, including immune regulation, barrier preservation, modulation of oxidative stress, and vascular stabilization. Therefore, disease‐specific sections below focus primarily on the evidence unique to each condition.

**TABLE 3:** Preclinical ocular disease models evaluating short‐chain fatty acid interventions.

Retinal disease	Animal model	SCFA utilized	Administration route and dosage	Findings	References
Bacterial endophthalmitis	*Staphylococcus aureus*endophthalmitis mouse	Sodium butyrate/phenylbutyrate (PBA)	Intravitreal injection of 10 μg of NaB/PBA	Reduced bacterial burden and retinal inflammation while preserving retinal function	(Singh et al. 2023)
Choroidal neovascularization/wet AMD model	Laser‐induced CNV mouse	Sodium butyrate	Intravitreal injection of 1‐5 mM NaB	Reduced CNV lesion size and angiogenesis	(Xiao et al. 2020)
Choroidal neovascularization/wet AMD model	Laser‐induced CNV mouse	Sodium butyrate	Intravitreal injection of 100 μg NaB	Reduced CNV size by 21% at Day 7 post‐laser	(Lyzogubov et al. 2020)
Corneal dysbiosis/homeostasis disorder	Antibiotic‐induced gut dysbiosis mouse	Acetate/propionate/butyrate	Drinking water containing 67.5 mmol/L NaA, 25.9 mmol/L NaP, or 40 mmol/L NaB	Restored corneal homeostasis disrupted by gut dysbiosis	(Liu et al. 2025)
Diabetic retinal injury/T2D	db/db mouse	Butyrate	Drinking water containing 5 g/L NaB	Improved systemic and retinal outcomes in early Type 2 diabetes	(Gong et al. 2025)
Diabetic retinopathy	STZ‐induced diabetic mouse	Sodium butyrate	500 mg/kg NaB via gavage	Reduced retinal thinning and microglial activation; improved retinal function	(Huang et al. 2023)
Dry eye disease	Desiccating stress mouse model	Tributyrin/Butyrate prodrug/precursor	Oral gavage (via drinking water) of 100 μL of 0.5 mM tributyrin	Reduced ocular‐surface inflammation in vivo and improved tear‐film homeostasis	(Schaefer et al. 2022)
Herpes simplex ocular disease	HSV‐1 ocular infection mouse	Sodium propionate	Drinking water containing 500 mM NaP	Reduced ocular lesion severity and inflammatory responses	(Sumbria et al. 2021)
Infectious keratitis/corneal inflammation	TLR ligand‐induced keratitis mouse	Sodium butyrate	Topical application of 2 μL of 0.5 or 1 mM NaB	Reduced corneal inflammatory responses through GPR43 signaling	(Wu et al. 2024)
Meibomian gland dysfunction (MGD)‐like ocular surface disease	ApoE^−/−^ mouse	Sodium butyrate	Topical application of 1, 5, or 10 mM NaB	Reduced MGD‐associated inflammation and modulated ocular‐surface microbiome	(Chen et al. 2025)
Retinopathy of Prematurity	Oxygen‐induced Retinopathy (OIR)	Sodium butyrate	Intraperitoneal injection of ~200‐500 mg/kg per day during hyperoxia and succeeding hypoxia window	Sodium butyrate supplementation significantly protects against pathological angiogenesis	(Oxenrider et al. 2025)
Uveitis	Experimental autoimmune uveitis mouse	Sodium butyrate	Oral administration of 1 g/kg/time point of NaB	Reduced ocular inflammation and shifted Th17/Treg balance	(Chen et al. 2017)
Uveitis	Experimental autoimmune uveitis mouse	Acetate/propionate/butyrate	Oral administration of 300 mM NaB, 300 mM NaA, or 150 or 300 mM NaP	Propionate and butyrate showed the most benefit; reduced uveitis severity	(Nakamura et al. 2017)
Uveitis	Endotoxin‐induced uveitis mouse	Acetate/butyrate	Intraperitoneal injection of 4 mg of NaA or 500 mg/kg NaB	Reduced inflammatory‐cell infiltration and inflammatory cytokines	(Chen et al. 2021)
Zika virus‐associated ocular disease	ZIKV‐infected mouse	Sodium acetate/sodium butyrate/phenylbutyrate	Intraperitoneal injection of 100 mg kg of NaB/PBA or NaA	Acetate and butyrate‐related treatments reduced ZIKV‐associated inflammation and retinal pathology	(Deshmukh et al. 2026)

Abbreviations: AMD, age‐related macular degeneration; ApoE, apolipoprotein E; CNV, choroidal neovascularization; GPR43, G protein–coupled receptor 43; HSV‐1, herpes simplex virus type 1; MGD, meibomian gland dysfunction; NaA, sodium acetate; NaB, sodium butyrate; NaP, sodium propionate; OIR, oxygen‐induced retinopathy; PBA, phenylbutyrate; SCFA, short‐chain fatty acid; STZ, streptozotocin; T2D, type 2 diabetes; Th17, T helper 17 cells; TLR, Toll‐like receptor; Treg, regulatory T cells; ZIKV, Zika virus.

#### 4.2.1 Immune‐Mediated Ocular Inflammation

Immune‐mediated ocular inflammation provides the most robust and consistent evidence linking SCFAs to disease. In experimental autoimmune uveitis, oral SCFA administration significantly reduces disease severity, accompanied by alterations in systemic immune responses and reduced migration of autoreactive lymphocytes to the eye (Nakamura et al. 2017; Horai et al. 2015). These findings provide compelling evidence that gut‐derived metabolites can influence ocular immune responses through systemic immune regulation. Complementary findings in endotoxin‐induced uveitis demonstrate reduced intraocular inflammation and cytokine production following SCFA treatment, alongside detection of these metabolites within ocular tissues (Nakamura et al. 2017; Chen et al. 2021; Chen et al. 2017). In dry eye disease, human studies report alterations in gut microbiome composition, including reduced microbial diversity and changes in taxa associated with immune regulation (Moon et al. 2020; Mendez et al. 2020). These findings are consistent with pathways influenced by microbiome‐derived metabolites, although direct evidence linking SCFAs to disease severity remains limited. Supporting preclinical data indicate that oral tributyrin reduces ocular surface inflammation and that the SCFA transporter SLC5A8 is expressed in the corneal and conjunctival epithelia (Schaefer et al. 2022). SCFA signaling has also been implicated in corneal inflammatory disease. In microbial keratitis models, SCFAs suppress corneal inflammatory responses, with evidence supporting an anti‐inflammatory role for FFAR2/GPR43 signaling (Wu et al. 2024). These findings extend the functional relevance of SCFAs beyond intraocular inflammation to ocular surface pathology (Table 3).

#### 4.2.2 Diabetic Retinopathy

Evidence for SCFA involvement in diabetic retinopathy is derived primarily from preclinical animal studies, with limited but growing support from human metabolomic investigations. In diabetic mouse models, sodium butyrate administration improves retinal vascular and neural integrity and modulates systemic metabolic and inflammatory pathways (Huang et al. 2023; Puddu et al. 2014; Gong et al. 2025).

Clinical studies in patients with diabetes demonstrate alterations in gut microbiome composition and circulating metabolites consistent with impaired microbial fermentation (Qin et al. 2025; Karlsson et al. 2013). Although direct quantification of SCFAs within ocular compartments is not yet available, systemic alterations suggest that SCFA‐related pathways may contribute to disease progression. Early retinal alterations associated with hyperglycemia may also be influenced by SCFA signaling. Experimental studies demonstrate that butyrate supplementation can mitigate retinal dysfunction under metabolic stress (Huang et al. 2023), suggesting that SCFAs may exert protective effects during early disease stages preceding overt microvascular damage (Table 3).

Several limitations should be considered when interpreting these findings. Most mechanistic studies have been conducted in rodent models, which do not fully recapitulate the complexity and heterogeneity of human diabetic retinopathy. Furthermore, reported associations between altered SCFA levels and disease severity may be confounded by dietary patterns, glycemic control, obesity, medication use, and other metabolic factors known to influence gut microbiome composition. Consequently, current evidence supports an association between SCFA dysregulation and diabetic retinopathy but remains insufficient to establish a direct causal role in humans.

#### 4.2.3 Age‐Related Macular Degeneration

Current evidence in AMD remains largely observational and is based predominantly on microbiome profiling and metabolomic association studies rather than direct mechanistic or interventional investigations. Patients with AMD exhibit differences in microbial communities and predicted metabolic pathways associated with microbial fermentation (Zinkernagel et al. 2017; Luo and Skondra 2023). More recently, metabolomic analyses have identified alterations in microbial‐derived metabolites in both fecal and circulating compartments (Parekh et al. 2025). These findings are consistent with broader disruptions in gut‐derived metabolic networks, particularly in non‐neovascular AMD, where chronic inflammation and metabolic dysfunction are prominent features. However, direct measurement of SCFAs within ocular tissues remains lacking, and current studies primarily infer their involvement. Interpretation of AMD‐associated microbiome studies should be approached with caution. Reported microbial signatures are not always reproducible across cohorts, and conflicting findings have been observed regarding the specific taxa associated with disease progression. Moreover, most studies infer altered SCFA production through microbial composition rather than direct metabolite quantification. As a result, whether SCFA deficiency is a driver of disease, a consequence of disease‐related physiological changes, or merely a biomarker of altered gut ecosystem function remains unresolved. Hence, SCFAs are best considered potential contributors within the gut‐retina axis rather than established mediators or drivers of AMD pathogenesis (Table 3). At present, available data are insufficient to support SCFA‐targeted interventions in AMD, and additional mechanistic and interventional studies are required.

#### 4.2.4 Pathological Neovascularization

Direct evidence linking SCFAs to ocular angiogenesis has historically been limited. Microbiome‐based studies demonstrate that alterations in gut microbial composition can influence angiogenic responses in retinal disease models, suggesting a role for microbiota‐derived metabolites in modulating retinal vascular pathology (Zinkernagel et al. 2017; Andriessen et al. 2016; Trompette et al. 2014). In non‐ocular systems, SCFAs regulate endothelial function, inflammatory signaling, and angiogenesis‐related pathways, including those mediated by VEGF, providing mechanistic plausibility for their role in vascular remodeling (Zinkernagel et al. 2017; Robles‐Vera et al. 2020; Guo et al. 2023).

Importantly, emerging preclinical studies have provided initial evidence addressing this gap in ocular systems. A recent study by our research group demonstrated that oral administration of sodium butyrate significantly attenuates pathological retinal neovascularization in the oxygen‐induced retinopathy (OIR) model, a well‐established model of ischemia‐driven retinal angiogenesis. This work further showed that butyrate modulates not only vascular pathology but also neuronal and microglial responses, highlighting its multifaceted role in retinal disease processes (Oxenrider et al. 2025). Complementing these findings, Xiao et al. demonstrated that sodium butyrate inhibits neovascularization, at least in part, by suppressing the TXNIP/VEGFR2 signaling pathway, providing mechanistic evidence that butyrate can directly modulate VEGF‐driven angiogenic signaling (Xiao et al. 2020).

Additional support for the anti‐angiogenic effects of SCFAs comes from studies in alternative ocular neovascularization models. Lyzogubov et al. reported that sodium butyrate significantly reduces choroidal neovascularization in a laser‐induced model, further extending its anti‐angiogenic efficacy beyond retinal ischemia models (Lyzogubov et al. 2020). This study suggests the possible therapeutic utility of butyrate in experimental ocular neovascularization models, compared with other anti‐angiogenic and anti‐inflammatory agents, reinforcing its role as a modulator of pathological ocular angiogenesis (Table 3).

These findings provide preliminary experimental support for the hypothesis that SCFAs may modulate pathological angiogenesis within the retina. Together with prior mechanistic insights from non‐ocular systems, these findings suggest that microbiota‐derived metabolites could contribute to pathways involved in neurovascular dysfunction in retinal diseases. Nevertheless, several uncertainties remain. The majority of available data are derived from preclinical models, and the extent to which these findings translate to human retinal vascular diseases remains unknown. Furthermore, the relative contribution of direct retinal SCFA signaling versus systemic immunometabolic effects has not been clearly established. While butyrate consistently exhibits anti‐angiogenic effects in current studies, evidence regarding acetate and propionate in ocular angiogenesis remains limited, highlighting an important area for future investigation. However, further validation across multiple ocular angiogenesis models, including laser‐induced choroidal neovascularization, will be necessary to fully define the therapeutic potential and underlying mechanisms of SCFA‐mediated vascular regulation.

#### 4.2.5 Glaucoma and Retinal Ganglion Cell Degeneration

Evidence linking SCFAs to glaucoma is currently limited to observational human studies and microbiome analyses, with relatively little mechanistic or interventional validation. Human studies have identified altered gut microbiome profiles in glaucoma, including a reduced abundance of SCFA‐producing bacteria (Krilis et al. 2024; Ullah et al. 2024; Vergroesen et al. 2024). This pattern is consistent with a possible association between microbiome‐derived metabolic activity and neuroinflammatory processes. At present, however, direct evidence demonstrating that SCFA modulation influences retinal ganglion cell survival, intraocular pressure, or optic nerve degeneration is limited. SCFAs are therefore best viewed as candidate modulators of neuroinflammation and metabolic stress within glaucoma rather than established therapeutic targets (Ullah et al. 2024; Liu et al. 2023). Consequently, any therapeutic implications of SCFA modulation in glaucoma should currently be considered speculative.

#### 4.2.6 Emerging Retinal Degenerative and Infectious Ocular Contexts

In inherited retinal degeneration, shifts in gut microbiome composition have been associated with disease progression, although current evidence does not establish SCFAs as direct mediators (Kutsyr et al. 2021). However, emerging studies across infectious and inflammatory ocular models provide increasing evidence for SCFA‐mediated immunoregulation. Infectious ocular disease represents a developing area, with findings indicating that butyrate and acetate can inhibit viral replication and associated ocular pathology through FFAR2/GPR43 signaling (Deshmukh et al. 2026), while dietary supplementation with sodium propionate suppresses viral immuno‐inflammatory lesions, further highlighting the antiviral and anti‐inflammatory potential of SCFAs (Sumbria et al. 2021). In bacterial infections, butyrate enhances host defense by promoting autophagy and attenuating inflammatory responses, suggesting a role in controlling intraocular immune activation (Singh et al. 2023).

Beyond infectious contexts, SCFAs also contribute to the maintenance of ocular surface and tissue homeostasis. Restoration of corneal integrity following antibiotic‐induced gut dysbiosis has been linked to SCFA supplementation, supporting a functional gut‐eye axis (Liu et al. 2025). Additionally, sodium butyrate has been shown to modulate the ocular surface microbiome and reduce inflammation in meibomian gland dysfunction, reinforcing its role in microbiome‐dependent immune regulation (Chen et al. 2025). Across ocular disease contexts, evidence follows a spectrum of support: immune‐mediated and infectious conditions show strong experimental validation, while metabolic, degenerative, and angiogenic diseases are supported by a combination of preclinical and associative human data. Collectively, these findings are consistent with the possibility that SCFAs may act as systemic modulators of shared pathological processes—particularly inflammation, immune dysregulation, and microbiome imbalance—rather than as disease‐specific effectors. Similarly, the therapeutic relevance of SCFAs in inherited retinal degeneration remains uncertain due to the limited mechanistic evidence currently available.

### 4.3 Observational Human, Metabolomic, and Emerging Clinical Evidence

Human evidence linking SCFAs to ocular disease is derived primarily from observational microbiome and metabolomic studies, with comparatively limited interventional data. As such, current findings remain largely associative and hypothesis‐generating.

#### 4.3.1 Microbiome‐Based Clinical Associations

Clinical studies consistently report gut dysbiosis in patients with ocular diseases, including diabetic retinopathy, AMD, uveitis, and dry eye disease. These alterations frequently involve reduced abundance of SCFA‐producing taxa such as *Faecalibacterium*, *Roseburia*, and *Bacteroides* (Huang et al. 2023; Moon et al. 2020; Zinkernagel et al. 2017; Krilis et al. 2024). In diabetic retinopathy, alterations in the microbiome are associated with systemic inflammation and endothelial dysfunction (Beserra et al. 2015). Studies examining the gut–retina axis have further shown that reduced SCFA production correlates with inflammatory and metabolic processes central to retinal disease progression (Ciurariu et al. 2025; Huang et al. 2023; Robles‐Vera et al. 2020). In uveitis, microbiome‐associated immune dysregulation may link microbial metabolites to ocular inflammation (Nguyen et al. 2024).

#### 4.3.2 Metabolomic Evidence and SCFA Quantification

Metabolomic profiling provides more direct insight into microbial metabolic activity in ocular disease. In diabetic retinopathy, circulating levels of SCFAs, including acetate and butyrate, are significantly reduced and correlate with immune and inflammatory biomarkers (Qin et al. 2025). Ophthalmic metabolomics studies also reveal broad metabolic alterations across ocular diseases, including pathways related to lipid metabolism, oxidative stress, and energy metabolism (Dmuchowska et al. 2025). Although SCFA‐specific measurements in ocular tissues remain limited, advances in tear and aqueous humor metabolomics are improving the feasibility of localized metabolite analysis (Serrano‐Marin et al. 2025).

#### 4.3.3 Interventional and Translational Evidence

Interventional studies in humans remain limited but suggest that microbiome‐targeted approaches may influence ocular outcomes. Dietary strategies that promote SCFA production, including high‐fiber intake, are associated with improved systemic immune regulation and may indirectly impact ocular disease processes (Nguyen et al. 2024). Integrated human–animal studies provide additional translational support. In diabetic retinopathy, acetate supplementation improves retinal structure and reduces inflammatory responses in experimental models, supporting mechanisms suggested by clinical observations (Qin et al. 2025).

### 4.4 Contradictory Findings and Evidence Gaps

While many studies support a protective role for SCFAs, the available evidence is not uniformly consistent across ocular disease contexts. Several experimental reports suggest that SCFA effects may vary according to concentration, tissue microenvironment, disease stage, and receptor expression patterns. For example, whereas butyrate generally exhibits anti‐inflammatory and barrier‐protective properties, HDAC inhibition can influence angiogenic pathways in ways that may vary by cell type and physiological context. In retinal neovascularization models, sodium butyrate reduced pathological angiogenesis in both OIR and laser‐induced CNV; however, the magnitude of its effect varied across models (Oxenrider et al. 2025; Xiao et al. 2020). Findings from human studies are also less consistent. Although studies have identified altered gut microbial profiles in AMD, the taxa associated with disease differ across cohorts and disease stages. For example, Zinkernagel et al. reported enrichment of *Anaerotruncus, Oscillibacter, Ruminococcus torques, and Eubacterium ventriosum* in neovascular AMD, whereas Parekh et al. identified increased *Desulfovibrionales and Terrisporobacter* in advanced AMD and directly measured lower fecal acetate, butyrate, and propionate (Zinkernagel et al. 2017; Luo and Skondra 2023; Parekh et al. 2025). Differences in diet, metabolic status, medication use, disease stage, sequencing methods, and analytical approaches may contribute to these variations. Moreover, most studies infer SCFA production from microbial composition rather than directly measuring SCFA concentrations in circulation or ocular tissues. Therefore, microbial composition alone may not accurately reflect SCFA availability or biological activity within the eye. The experimental evidence is still concentrated in a small number of animal models and is largely focused on butyrate. The effects of acetate and propionate in retinal disease remain much less clear. It also remains unresolved whether SCFAs act directly within retinal tissues or primarily through systemic immune and metabolic pathways. These findings indicate that SCFAs are plausible contributors to ocular disease pathways, but their causal role and therapeutic relevance in human disease have not been established. Direct measurement of SCFAs in ocular tissues, longitudinal studies, and well‐controlled interventional studies will be important to distinguish causal mechanisms from disease‐associated microbial changes.

## 5 Therapeutic Potential and Challenges

While growing interest in the gut‐eye axis has generated multiple potential therapeutic strategies, it is important to recognize that most evidence remains preclinical and proof‐of‐concept in nature. Direct clinical evidence supporting microbiome‐targeted or SCFA‐based interventions for retinal diseases is currently limited, and many proposed therapeutic approaches remain at an early stage of development. Therefore, the interventions discussed below should be viewed as emerging research strategies rather than established therapeutic modalities.

### 5.1 Therapeutic Strategies

Although encouraging results have been reported in several experimental models, the majority of studies evaluating dietary modulation, probiotics, synbiotics, FMT, or direct SCFA supplementation have been conducted in animal models or non‐ocular disease settings. The relevance of these findings to human retinal diseases remains to be fully established (Table 4).

**TABLE 4:** Potential therapeutic interventions targeting the SCFA axis in various diseases.

Interventions	Primary target	Evidence base	Pros	Cons	Potential ocular applications
Dietary fiber enrichment	Endogenous SCFA production via colonic fermentation	In vivo (mouse), epidemiologic, limited interventional clinical studies	High safety profile Physiologic regulation of SCFA gradients Broad metabolic benefits	Inter‐individual microbiome variability Slow onset Limited control over specific SCFA species	Potential risk‐modification strategy for DR and AMD; experimental relevance to ROP and retinal inflammation
Prebiotics (e.g., inulin, FOS, resistant starch)	Selective enrichment of beneficial SCFA‐producing taxa (e.g., *Bifidobacterium, Faecalibacterium, Roseburia* and related microbial guilds., *Firmicutes*)	In vivo, mechanistic, small clinical trials	Greater specificity than fiber Non‐invasive Scalable	Variable responder phenotypes GI intolerance at higher doses Indirect SCFA control	Experimental modulation of DR‐associated inflammation and metabolic dry eye disease
Probiotics (single or multi)	Microbial composition, barrier integrity, and indirect enhancement and SCFA‐producing capacity	In vivo, early clinical, mechanistic	Targeted microbial modulation Relatively rapid microbiome shifts	Colonization instability Strain‐specific effects Regulatory variability	Experimental Dry eye therapy; potential adjunctive therapy in DR
Synbiotics	Alleviating dysbiosis within the gut microbiome and SCFA production	Preclinical, mechanistic and limited clinical evidence	Protects against systemic inflammation; restored gut microbiome balance; increases SCFA abundance	Unstable probiotic viability; strain and formulation‐specific effects; limited human studies	Experimental ocular surface disease, uveitis, glaucoma, and systemic inflammatory conditions affecting ocular health
Direct SCFA supplementation (acetate, propionate, butyrate)	Systemic SCFA exposure; GPCR signaling (GPR41/43/109A)	In vivo, mechanistic	Rapid pharmacologic effect Bypasses microbiome dependency	Short half‐life PK variability Tolerability issues (odor, GI effects) Rapid intestinal absorption limits distal delivery	Acute inflammatory modulation; experimental DR or AMD models
Tributyrin (butyric acid)	Sustained systemic butyrate delivery	In vivo, emerging translational studies	Improved bioavailability Reduced odor Controlled release Improved oral tolerability compared with free butyrate	Limited clinical data Dose optimization unresolved	ROP prevention; pathological angiogenesis in DR
Combination approaches (fiber + probiotic)	Multi‐level modulation of production and signaling	In vivo, mechanistic, growing translational evidence	Synergistic effects Resilience against microbiome variability	Increased complexity Adherence challenges Interaction effects	Long‐term prevention strategies in DR and AMD
Fecal Microbiome Transplantation	Transplantation of a fecal microbiome from a healthy donor into a dsybiotic host	In vivo (mouse), and translational studies (human)	Restored gut microbiome homeostasis; reduced inflammation; improved gut barrier integrity; enrichment of SCFA‐producing taxa	Risk of pathogen transmission, donor‐dependent variability, and uncertain long‐term safety	Experimental strategy for gut‐retina axis modulation in AMD, DR, uveitis, and ocular surface disease

Abbreviations: AMD, age‐related macular degeneration; DR, diabetic retinopathy; FOS, fructooligosaccharides; GI, gastrointestinal; GPCR, G protein–coupled receptor; GPR109A, G protein–coupled receptor 109A; GPR41, G protein–coupled receptor 41; GPR43, G protein–coupled receptor 43; NaB, sodium butyrate; PK, pharmacokinetic; ROP, retinopathy of prematurity; SCFA, short‐chain fatty acid.

#### 5.1.1 Dietary Fiber

Dietary fiber is a crucial component of a balanced diet, primarily found in fruits, vegetables, legumes, and whole grains (Kumar et al. 2020; Annunziata et al. 2020). Dietary fiber promotes microbial fermentation and endogenous SCFA production, representing a physiological approach to microbiome modulation.

**FIGURE 4:**
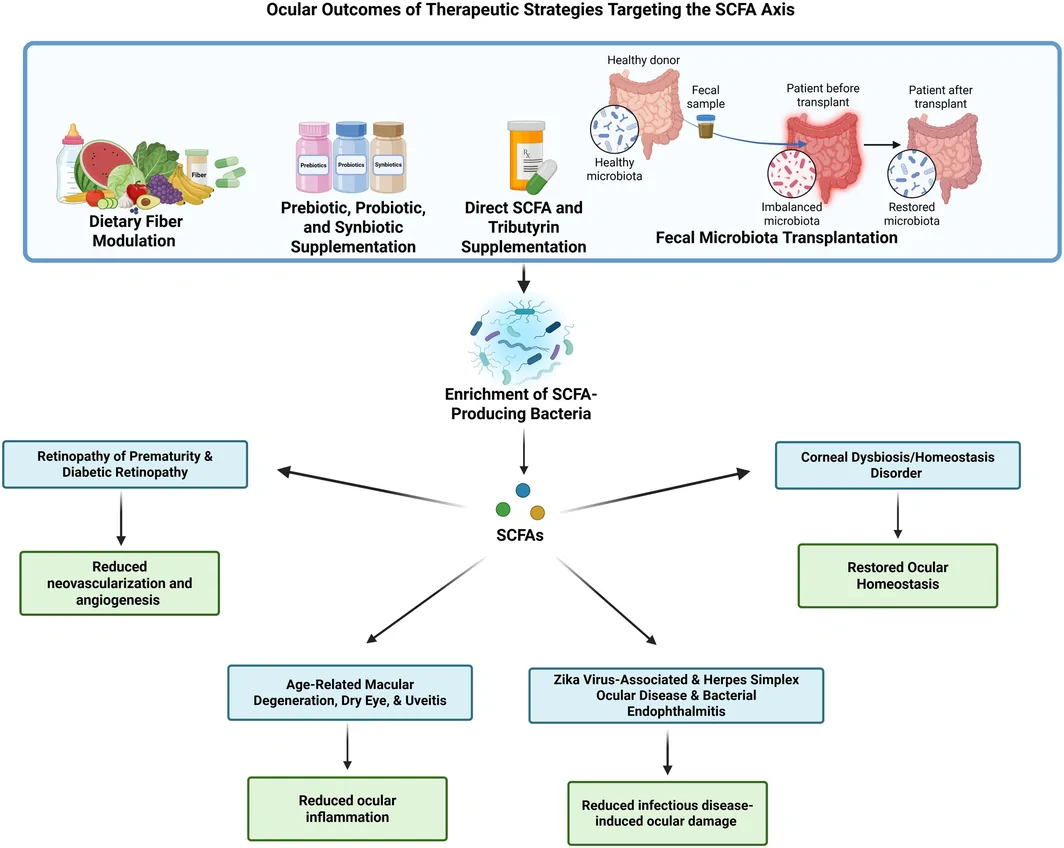
Therapeutic strategies targeting the short‐chain fatty acid (SCFA) axis to improve ocular outcomes. Conceptual schematic illustrating therapeutic approaches aimed at modulating the gut microbiome–SCFA axis to promote ocular health. Dietary modulation, including increased intake of fiber‐rich and microbiome‐supportive foods, shapes gut microbial composition and enhances endogenous production of SCFAs, particularly by butyrate‐producing bacteria. Targeted microbiome interventions may further enrich SCFA‐producing taxa. In parallel, direct supplementation with SCFAs or SCFA prodrugs such as tributyrin provides an exogenous strategy to elevate systemic SCFA availability. These complementary approaches converge on improved ocular outcomes, including reduced ocular inflammation, stabilization of vascular integrity, and enhancement of ocular surface and retinal homeostasis, highlighting the translational potential of targeting the SCFA axis in retinal and ocular diseases. The illustration was created using BioRender.com

Evidence from numerous clinical studies indicates that a high‐fiber diet can increase microbial diversity, thereby improving gut barrier function and reducing the risk of conditions such as irritable bowel syndrome and even certain metabolic disorders (Sanna et al. 2019; Caetano and Castelucci 2022). However, there are limitations to the therapeutic use of dietary fiber (Table 3). Not all types of fiber are equally beneficial, as their effects can vary significantly depending on an individual's gut microbiota composition and the presence of specific microbial species (Liu et al. 2018; Xie and Lin 2025). Additionally, excessive fiber intake can lead to gastrointestinal discomfort, such as bloating and gas, making it imperative for healthcare providers to tailor fiber recommendations to individual patient needs (Ciurariu et al. 2025; Scott et al. 2020) (Figure 4).

#### 5.1.2 Pre/Probiotics and Synbiotics

Prebiotics and probiotics represent another pivotal strategy in modulating gut health and, by extension, the gut–eye axis (Table 3). Prebiotics, typically non‐digestible food ingredients like inulin and fructooligosaccharides, serve as substrates that stimulate the growth and/or activity of beneficial microbial species (Ciurariu et al. 2025; Kang and Zivkovic 2021; Moles and Otaegui 2020). Probiotics, on the other hand, are live microorganisms that confer health benefits when consumed in adequate amounts (Kang and Zivkovic 2021; Moles and Otaegui 2020). The primary objective of these interventions is to selectively modify microbial composition and metabolic activity, including enhancement of SCFA‐producing taxa (Beukema et al. 2020; Kang and Zivkovic 2021; Cui et al. 2025). Despite this promising evidence, challenges remain, particularly regarding product consistency and strain specificity. The effectiveness of pre‐ and probiotics can vary widely depending on the specific strains used, the dosage, and the duration of administration (Ciurariu et al. 2025; Dang et al. 2021; Matar et al. 2024; Mao et al. 2025). Furthermore, the individual response can be highly variable, influenced by factors such as baseline microbiota composition and dietary habits (Ciurariu et al. 2025; Dang et al. 2021; Matar et al. 2024; Mao et al. 2025).

Synbiotics, a combination of prebiotics and probiotics, represent an advanced approach to microbiome modulation by synergistically enhancing the survival, colonization, and functional activity of beneficial microorganisms in the gut (Table 3). By providing both the live microbial strains (probiotics) and the specific substrates that selectively promote their growth (prebiotics), synbiotics aim to optimize microbial balance more effectively than either component alone. This dual mechanism has been shown to improve intestinal barrier integrity, increase production of beneficial metabolites such as SCFAs, and modulate systemic immune responses (Baba et al. 2025; Ma, Lian, et al. 2025), processes that are highly relevant to the gut–eye axis and retinal immune homeostasis (Alfuzaie 2023; Zhou et al. 2026).

Emerging evidence suggests that synbiotics may provide greater microbiome remodeling and SCFA enrichment than either prebiotics or probiotics alone. However, optimal strain‐substrate combinations, dosing regimens, and long‐term efficacy remain uncertain (Figure 4). These effects include downregulation of pro‐inflammatory cytokines, improved epithelial barrier function, and restoration of microbial diversity, all of which are implicated in retinal diseases such as AMD and diabetic retinopathy. Furthermore, synbiotic formulations may help overcome some limitations associated with probiotics alone, including poor microbial engraftment and variability in host response, by creating a more favorable ecological niche for administered strains. However, as with pre‐ and probiotics, challenges remain regarding optimal formulation, strain–substrate pairing, dosing strategies, and inter‐individual variability, necessitating further well‐controlled clinical studies to establish their therapeutic potential in ocular and systemic diseases.

#### 5.1.3 Fecal Microbiota Transplantation

Fecal microbiota transplantation (FMT) has emerged as a promising therapeutic strategy for restoring gut microbial homeostasis and modulating systemic inflammation in a range of diseases associated with dysbiosis (Hou et al. 2025; Ma, Zhang, et al. 2025; Karimi et al. 2024). By transferring a complex and functionally intact microbial community from healthy donors, FMT can reshape host microbial composition, enhance intestinal barrier integrity, and suppress pro‐inflammatory signaling pathways. These effects are particularly relevant to the gut–eye axis, where microbiota‐derived immune and metabolic mediators influence retinal homeostasis and disease progression (Alfuzaie 2023; Zhou et al. 2026).

Preclinical studies provide compelling evidence for the role of FMT in modulating retinal and neuroinflammatory processes. In a study by Parker et al., fecal microbiota transfer between young and aged mice demonstrated a direct causal relationship between gut microbial composition and age‐associated inflammation in distal organs, including the retina. Transfer of microbiota from aged donors into young mice induced retinal inflammation characterized by increased complement activation and pro‐inflammatory cytokine signaling, whereas transplantation of microbiota from young donors into aged mice reversed these changes, reducing inflammatory markers and restoring aspects of retinal homeostasis (Parker et al. 2022). These findings suggest that gut microbiota remodeling may directly influence retinal immune responses and functional integrity through systemic immune modulation and metabolite signaling.

Beyond retinal pathology, FMT has shown broad anti‐inflammatory effects in multiple preclinical disease models. For instance, FMT has been shown to alleviate metabolic and inflammatory phenotypes in mouse models of fatty liver disease, colitis, and atherosclerosis by restoring microbial diversity and reducing the expression of pro‐inflammatory cytokines, including IL‐1β and IL‐17. These systemic anti‐inflammatory effects are mechanistically linked to improved intestinal barrier function, reduced endotoxemia, and modulation of host immune pathways, all of which are relevant to retinal diseases driven by chronic low‐grade inflammation and vascular dysfunction (Mi et al. 2026).

Within the context of ocular disease, emerging clinical and translational evidence suggests that microbiota‐targeted interventions, including FMT, may have therapeutic potential (Figure 4). Although human studies remain limited, early interventional data indicate that modulating the gut microbiome with probiotics or FMT can reduce inflammatory ocular conditions such as uveitis, supporting the broader relevance of microbiota‐based therapies in eye health. Furthermore, recent reviews of the gut–retinal axis emphasize that microbial transplantation strategies may offer a novel avenue to influence disease trajectories in AMD, diabetic retinopathy, and other retinal disorders by modulating immune activation, complement pathways, and angiogenic signaling (Russell et al. 2023; Schiavone et al. 2025).

Although data in infants are still emerging, early‐life modulation of the gut microbiome is of particular interest given the critical window for microbiome establishment and immune system development. Disruptions in early microbial colonization are associated with pediatric eye conditions such as retinopathy of prematurity. This indicates that microbiota‐targeted interventions‐potentially involving FMT in controlled environments‐could provide lasting advantages by reestablishing microbial balance and reducing inflammation‐related programming (Chen et al. 2026).

Collectively, current evidence supports the concept that FMT can modulate systemic and retinal inflammation through the gut–eye axis (Table 3). While preclinical findings are highly encouraging, further well‐designed clinical studies are necessary to determine the safety, efficacy, and mechanistic specificity of FMT in retinal diseases. Nonetheless, microbiome‐based therapies hold significant promise as adjunctive strategies for targeting inflammation‐driven retinal pathologies.

#### 5.1.4 SCFA Supplementation

SCFA supplementation is emerging as a potential therapeutic approach to harness the beneficial effects of metabolites produced by the gut microbiota (Rios‐Covian et al. 2016; Berni Canani et al. 2012). Direct SCFA supplementation offers a strategy to bypass microbiome variability and increase systemic metabolite exposure (Dang et al. 2021; Moffett et al. 2020).

Evidence supporting SCFA supplementation indicates its potential to reduce symptoms of inflammatory bowel diseases, enhance gut barrier function, and exhibit neuroprotective properties (Facchin et al. 2024; He et al. 2020; Franzin et al. 2021), which may be relevant to the gut‐eye axis (Figure 4, Table 3). Nevertheless, SCFA supplementation has limitations. While SCFAs are generally considered safe and well‐tolerated, potential side effects, particularly at higher doses or with supplementation, may include gastrointestinal discomfort such as bloating, abdominal pain, gas, and altered bowel habits. These effects are likely related to luminal accumulation and fermentation within the gastrointestinal tract and are commonly observed in studies involving SCFA or fermentable fiber intake (Xiong et al. 2022). Additionally, the long‐term safety of SCFA supplementation remains to be fully elucidated.

A major translational challenge for SCFA supplementation is achieving therapeutically relevant exposure in ocular tissues. Following oral administration, a substantial proportion of SCFAs undergo rapid intestinal absorption and first‐pass hepatic metabolism, potentially limiting systemic and ocular bioavailability. Consequently, circulating concentrations may not accurately reflect tissue exposure within the retina or other ocular compartments. Development of optimized formulations, including sustained‐release prodrugs, encapsulated delivery systems, and synthetic SCFA analogs with improved pharmacokinetic profiles, may be necessary to overcome these limitations and enhance therapeutic utility in ophthalmology.

### 5.2 Key Challenges

As we explore the therapeutic potential of the aforementioned strategies, several key challenges arise. One of the principal barriers to clinical translation involves incomplete understanding of SCFA pharmacokinetics (PK) and pharmacodynamics (PD). Because SCFAs are rapidly absorbed and extensively metabolized by intestinal epithelial cells and the liver, systemic exposure is highly variable, and tissue‐specific bioavailability remains poorly defined (Dong and Cui 2022). Importantly, the lack of studies that quantified SCFA concentrations in ocular tissues after dietary, microbial, or pharmacological interventions makes it difficult to establish exposure‐response relationships relevant to retinal disease.

Delivery barriers also pose a significant challenge. Many therapeutic interventions aimed at modulating the gut microbiota do not effectively reach their target sites due to the selective nature of the gut environment and the effects of digestion (Mao et al. 2025; Guan and Liu 2023). This limitation emphasizes the need for innovative delivery systems that protect bioactive compounds from degradation and ensure they reach the intestines intact.

Ocular delivery presents additional challenges. While systemic administration is the most common approach in current studies, it remains unclear whether sufficient concentrations of SCFAs reach the retina, retinal pigment epithelium, or choroid to directly influence disease processes. Alternative approaches, including topical administration, periocular delivery, intravitreal injection, or the development of receptor‐selective agonists that mimic SCFA signaling, may provide more effective therapeutic strategies. However, these approaches remain largely unexplored.

Safety is another critical aspect that must be carefully considered. While pre/probiotics and SCFA supplementation generally have good safety profiles, the potential for adverse effects should not be overlooked, especially in vulnerable populations, such as those with compromised immune systems or specific microbiota dysbiosis (Liu et al. 2018). Thorough evaluation of the safety profiles associated with these therapeutic strategies is essential before widespread implementation.

Lastly, personalized responses to these therapeutic strategies represent a significant challenge in achieving optimal outcomes. Individual variations in genetic makeup, existing microbiota composition, and lifestyle factors can lead to differing responses to dietary interventions, prebiotics and probiotics, and SCFA supplementation (Mao et al. 2025; Portincasa et al. 2022). To maximize therapeutic efficacy, a personalized approach is necessary, emphasizing tailored interventions that consider each individual's unique characteristics and health needs.

## 6 Knowledge Gaps and Future Directions

An important limitation of the current literature is the predominance of associative evidence. Although numerous studies report links among gut dysbiosis, altered SCFA‐producing microbial taxa, and ocular disease, relatively few investigations directly measure SCFA concentrations in ocular tissues or evaluate receptor‐specific signaling mechanisms in vivo or use longitudinal designs to determine whether SCFA alterations precede disease onset or progression. Consequently, many proposed mechanisms remain biologically plausible but incompletely validated. Although evidence supporting gut‐eye axis signaling continues to expand, several unresolved mechanistic and translational questions limit clinical implementation of SCFA‐based therapies. Many challenges related to bioavailability, dosing, and safety were discussed in Section 5; the discussion below focuses on additional knowledge gaps requiring investigation.

### 6.1 Ocular SCFA Pharmacology: Distribution, Targets, and Mechanisms

A fundamental gap in the field concerns the pharmacokinetics and pharmacodynamics of SCFAs in ocular compartments. Most mechanistic insights into SCFA signaling come from the intestinal, hepatic, or immune systems, where SCFAs act through G protein–coupled receptors (GPR41/FFAR3, GPR43/FFAR2, GPR109A), HDAC inhibition, and modulation of mitochondrial metabolism. Whether and to what extent these signaling axes operate in the retina, the retinal pigment epithelium (RPE), or the choroid remains largely unresolved.

Evidence suggests that circulating SCFAs can cross epithelial and endothelial barriers under certain conditions; however, the eye is protected by highly specialized barrier systems, including the blood–retinal barrier and the tight junction‐rich RPE. It remains unclear whether physiologic concentrations of gut‐derived SCFAs reach ocular tissues directly, act indirectly by reprogramming systemic immune cells that subsequently traffic to the eye, or are supplied locally by microbial or metabolic processes within ocular‐associated niches. Moreover, the expression profiles and functional relevance of SCFA receptors in distinct ocular cell types—photoreceptors, Müller glia, microglia, endothelial cells, pericytes, and RPE—remain incompletely characterized. Without this foundational knowledge, it is difficult to ascribe causal mechanisms to the protective or pathogenic associations observed in preclinical models. Notably, while receptor expression has been reported in several ocular cell types, most evidence remains descriptive. Functional receptor‐mapping studies employing cell‐specific deletion or pharmacological targeting strategies are largely lacking.

### 6.2 Optimal Dosing and Route of Administration

A second major gap concerns dose–response relationships and route specificity in SCFA‐based interventions. A major unresolved issue is defining therapeutically effective ocular exposure levels. Existing studies report protocols using widely different formulations, dosing schedules, and delivery routes, making comparisons across studies difficult and preventing establishment of standardized treatment paradigms. Importantly, SCFAs exhibit pleiotropic, concentration‐dependent effects, acting as anti‐inflammatory mediators at low concentrations while potentially promoting angiogenesis or metabolic stress responses at higher concentrations. The optimal dose may therefore vary by disease context, disease stage, and target cell population. For ocular diseases, additional complexity arises from the distinction between systemic exposure and local ocular delivery. Whether topical, intravitreal, or periocular delivery of SCFAs or synthetic analogs with improved stability can achieve therapeutic benefit without off‐target effects remains unexplored. Establishing robust pharmacokinetic models and dose‐ranging studies will be essential before advancing toward clinical translation.

Furthermore, equivalent exposure levels across oral, intraperitoneal, topical, and intravitreal routes of administration remain unknown. Establishing standardized dosing strategies and identifying pharmacodynamic biomarkers of target engagement will be important prerequisites for future clinical trials.

### 6.3 Long‐Term Safety

Although SCFAs are often considered intrinsically safe because of their endogenous origin, long‐term ocular safety cannot be assumed. Existing literature overwhelmingly focuses on short‐term exposures in systemic disease models, leaving critical questions about chronic administration, cumulative exposure, and interactions with established ocular therapies, such as anti‐VEGF agents or corticosteroids, unanswered. Given that SCFAs influence epigenetic regulation through HDAC inhibition, prolonged exposure could conceivably alter gene expression programs in slowly renewing ocular tissues, such as the RPE or retinal vasculature. Additionally, chronic modulation of immune tone, particularly microglial activation states, may have unintended consequences in aging retinas already predisposed to para‐inflammation and neurodegeneration. Longitudinal studies in aged animal models, with careful assessment of retinal structure, function, and inflammatory status, are therefore essential to define therapeutic windows and safety margins. Importantly, no studies have evaluated chronic SCFA administration in large‐animal ocular models or in long‐term human ophthalmic trials, representing a major barrier to translation.

Safety considerations may also differ according to route of administration. While oral SCFA supplementation has generally demonstrated favorable tolerability profiles, local ocular delivery strategies may present unique risks related to retinal toxicity, altered barrier function, or unintended modulation of resident immune cells. Systematic toxicology studies and long‐term assessments of retinal structure and function will therefore be necessary before consideration of clinical implementation.

### 6.4 Integration of Multi‐Omics Approaches

Another critical limitation in the current literature is the lack of integrated multi‐omics analyses linking gut microbiota composition, microbial metabolite production, and ocular outcomes. Most studies examine individual components—microbiome taxonomy, circulating SCFA levels, or retinal phenotypes—alone, precluding systems‐level inference.

Combining metagenomics, metabolomics, transcriptomics, and epigenomics across gut, blood, and ocular tissues would enable identification of causal pathways rather than merely associative correlations. Such approaches could reveal, for example, whether specific microbial consortia preferentially produce SCFAs that modulate retinal angiogenesis, or whether host genetic and epigenetic factors determine susceptibility to SCFA‐mediated effects. Importantly, these integrative datasets would also help reconcile discrepancies across studies and identify biomarkers predictive of therapeutic responsiveness. Such datasets would also help reconcile conflicting findings across microbiome studies, in which disease‐associated microbial signatures often vary substantially between cohorts.

### 6.5 Need for Rigorous Human Clinical Studies

Perhaps the most significant translational gap is the lack of well‐controlled human clinical trials directly evaluating SCFA‐based interventions for ocular disease. While preclinical models provide compelling proof of concept, extrapolation to human pathology is complicated by interindividual variability in microbiome composition, diet, metabolism, and immune aging. Future clinical studies must move beyond correlational microbiome analyses toward mechanistically informed trial designs that incorporate baseline microbial profiling, longitudinal metabolite measurements, and standardized ocular imaging endpoints. Including diverse patient populations across age, sex, metabolic status, and disease subtype will be essential to determine generalizability and identify subgroups most likely to benefit from intervention.

### 6.6 Toward Personalized and Precision‐Based Therapies

Ultimately, the therapeutic potential of SCFAs in ocular disease may lie in personalized medicine approaches that account for interindividual microbial and metabolic heterogeneity. Rather than uniform supplementation strategies, future interventions may involve targeted dietary modifications, selective microbial enrichment, or precision‐engineered SCFA analogs tailored to a patient's microbial and immunometabolic profile. Such strategies align with a broader shift toward precision ophthalmology, in which disease mechanisms are stratified not only by anatomical phenotype but also by systemic metabolic and immune context. As our understanding of the Gut–Eye Axis deepens, leveraging microbial metabolites such as SCFAs may offer novel opportunities to modulate retinal inflammation, angiogenesis, and tissue resilience in ways that complement existing therapies. By systematically addressing these knowledge gaps—from basic pharmacology and dosing to safety, systems biology, and personalized translation—the field can move beyond associative observations toward a mechanistic framework for microbial metabolite–based ocular therapeutics. Continued interdisciplinary investigation at the interface of microbiology, immunology, metabolism, and vision science will be essential to realize the full therapeutic promise of the Gut–Eye Axis and to advance innovative treatment strategies for retinal disease.

From a translational perspective, future development of SCFA‐based therapies will likely require integration of microbiome profiling, metabolomic monitoring, pharmacokinetic assessment, and advanced drug‐delivery technologies. Because interindividual variability in microbiome composition may substantially affect endogenous SCFA production and therapeutic responsiveness, precision‐medicine approaches may ultimately prove more effective than uniform supplementation strategies. Such efforts will be essential for transforming promising preclinical observations into clinically actionable therapeutic interventions.

## 7 Conclusion

Overall, available experimental and preclinical evidence suggests that gut microbiota‐derived SCFAs may contribute to gut‐eye axis signaling and influence biological processes relevant to ocular health and disease. Studies indicate that SCFAs can modulate inflammatory signaling, barrier integrity, immune cell trafficking, metabolic homeostasis, and retinal vascular responses. Experimental models further suggest that microbiome‐derived metabolites may influence ocular inflammation and pathological neovascularization. However, much of the current evidence remains preclinical, and human data are largely associative. Consequently, although SCFAs represent promising candidates for therapeutic investigation, additional mechanistic studies, pharmacokinetic characterization, and well‐controlled clinical trials are required to determine their translational relevance and therapeutic utility in ocular diseases.

## Author Contributions

Conceptualization: Aleah J. Brokemond and Ravirajsinh N. Jadeja. Software, analysis: Aleah J. Brokemond, Ronny Amamoo, Fapianey J. Alexandre, Sewedo B. Ajisegiri. Writing – original draft preparation: Aleah J. Brokemond and Ravirajsinh N. Jadeja. Writing – review and editing: Aleah J. Brokemond, Ronny Amamoo, Fapianey J. Alexandre, Sewedo B. Ajisegiri, Menaka C. Thounaojam, Pamela M. Martin, and Ravirajsinh N. Jadeja. Funding acquisition: Menaka C. Thounaojam, Pamela M. Martin, and Ravirajsinh N. Jadeja.

## Funding

The authors' laboratory is funded by the EY035336 grant and the Chan Zuckerberg Initiative (CZIF2022‐007043) accelerate precision health program to RNJ, EY034568 to MCT, and EY033264 to RNJ & PMM. Student trainees were supported by GM144927, HL007737, AI007281, and UC2GM162929.

## Ethics Statement

The authors have nothing to report.

## Consent

The authors have nothing to report.

## Conflicts of Interest

The authors declare no conflicts of interest.

## Data Availability

The authors have nothing to report.
